# Supplementary material for: Supersymmetry in the time domain and its applications in optics
Source: Nat Commun. 2020 Feb 10;11:813. doi: 10.1038/s41467-020-14634-0 (PMC7010821; doi:10.1038/s41467-020-14634-0)
Supplement: Supplementary file 1 — Supplementary information [file 41467_2020_14634_MOESM1_ESM.pdf]

Supplementary Information: Supersymmetry in the time domain and its applications in optics

Carlos García-Meca*,†, Andrés Macho Ortiz*,† and Roberto Llorente Sáez

Nanophotonics Technology Centre, Universitat Politècnica de València, Valencia 46022, Spain

∗corresponding author: cargarm2@ntc.upv.es, amachor@ntc.upv.es
†These authors have contributed equally to this work

Supplementary Note 1:
Temporal scattering – optical wave equation

Remarkably, the theory of temporal supersymmetry (T-SUSY) applied to temporal scattering is valid for dielectric (or magnetic), linear, anisotropic, heterogeneous, time-varying, and temporally non-dispersive media. As demonstrated in this section, these scenarios (including the particular situation in which a refractive index of the form given by equation (2) is assumed) lead to the temporal Helmholtz equation (3).

Let us start by considering the all-dielectric case ($\boldsymbol{\mu}_{\mathrm{r}}(\mathbf{r},t)=\mathbf{I}_3$). For such media, by combining Faraday's and Ampère's laws (applying the curl operator in Faraday's law and taking the time derivative of Ampère's law) it is straightforward to demonstrate that the *exact* time-domain vector wave equation for the electric flux density $\mathbf{D}$ takes the form:

$$-\nabla\times\nabla\times\left[\boldsymbol{\varepsilon}_{\mathrm{r}}^{-1}(\mathbf{r},t)\,\mathbf{D}(\mathbf{r},t)\right]=\frac{1}{c_0^2}\frac{\partial^2}{\partial t^2}\mathbf{D}(\mathbf{r},t)\,, \tag{1}$$

where $\boldsymbol{\varepsilon}_{\mathrm{r}}(\mathbf{r},t)$ is the medium relative permittivity tensor. Let us now assume that the relative permittivity can be expressed as:

$$\boldsymbol{\varepsilon}_{\mathrm{r}}(\mathbf{r},t)=\varepsilon_{\mathrm{T}}(t)\,\boldsymbol{\varepsilon}_{\mathrm{S}}(\mathbf{r})\,, \tag{2}$$

with $\boldsymbol{\varepsilon}_{\mathrm{S}}(\mathbf{r})$ being a tensor and $\varepsilon_{\mathrm{T}}(t)$ a scalar. It then follows that:

$$-\nabla\times\nabla\times\left(\boldsymbol{\varepsilon}_{\mathrm{S}}^{-1}(\mathbf{r})\,\mathbf{D}(\mathbf{r},t)\right)=\frac{1}{c_0^2}\varepsilon_{\mathrm{T}}(t)\,\frac{\partial^2}{\partial t^2}\mathbf{D}(\mathbf{r},t)\,. \tag{3}$$

The same wave equation applies to all-magnetic media ($\boldsymbol{\varepsilon}_{\mathrm{r}}(\mathbf{r},t)=\mathbf{I}_3$) if $\mathbf{D}$ is replaced by the magnetic flux density $\mathbf{B}$ and $\boldsymbol{\varepsilon}_{\mathrm{r}}(\mathbf{r},t)$ by the relative permeability tensor $\boldsymbol{\mu}_{\mathrm{r}}(\mathbf{r},t)=\mu_{\mathrm{T}}(t)\,\boldsymbol{\mu}_{\mathrm{S}}(\mathbf{r})$.

Applying separation of variables in the electromagnetic field under analysis $\mathbf{F}\in\{\mathbf{D},\mathbf{B}\}$:

$$\mathbf{F}(\mathbf{r},t)=\psi(t)\,\boldsymbol{\Phi}(\mathbf{r})\,, \tag{4}$$

Supplementary Equation 3 becomes:

$$-\nabla\times\nabla\times\left(\boldsymbol{\varepsilon}_{\mathrm{S}}^{-1}(\mathbf{r})\,\boldsymbol{\Phi}(\mathbf{r})\right)=\frac{\varepsilon_{\mathrm{T}}(t)}{c_0^2}\frac{\ddot{\psi}(t)}{\psi(t)}\boldsymbol{\Phi}(\mathbf{r})\,, \tag{5}$$

with $\ddot{\psi}(t)$ being the second-order time derivative of $\psi(t)$. Therefore, we must have:

$$\varepsilon_{\mathrm{T}}(t)\,\frac{\ddot{\psi}(t)}{\psi(t)}=C, \tag{6}$$

where C is a constant. Defining $n_{\mathrm{T}}^2(t):=\varepsilon_{\mathrm{T}}(t)$ (or $n_{\mathrm{T}}^2(t):=\mu_{\mathrm{T}}(t)$ in the all-magnetic case) and assuming that $n_{-}:=n_{\mathrm{T}}(t\to-\infty)$ is also a constant, we obtain $C=-\omega^2 n_{-}^2$ for a monochromatic wave with an angular frequency ω at $t\to-\infty$, yielding equation (3). For a polychromatic wave, the total field is given by the superposition of the solutions to Supplementary Equation 6 for each angular frequency.

In the particular case of *isotropic* all-dielectric media, Supplementary Equation 1 can be recast as (using $\nabla\cdot\mathbf{D}=0$):

$$\triangle\left(\frac{1}{n^2(\mathbf{r},t)}\mathbf{D}(\mathbf{r},t)\right)-\nabla\left(\nabla\left(\frac{1}{n^2(\mathbf{r},t)}\right)\cdot\mathbf{D}(\mathbf{r},t)\right)-\frac{1}{c_0^2}\frac{\partial^2}{\partial t^2}\mathbf{D}(\mathbf{r},t)=\mathbf{0}, \tag{7}$$

with $n^2(\mathbf{r},t)=\varepsilon_{\mathrm{r}}(\mathbf{r},t)$. The same equation applies to isotropic all-magnetic media with $n^2(\mathbf{r},t)=\mu_{\mathrm{r}}(\mathbf{r},t)$ and replacing $\mathbf{D}$ by $\mathbf{B}$. Obviously, proceeding as in the general case by applying separation of

variables to Supplementary Equation 7, equation (3) is again recovered. In the case of simultaneously dielectric and magnetic materials ($n^2(\mathbf{r},t) = \varepsilon_\mathrm{r}(\mathbf{r},t)\,\mu_\mathrm{r}(\mathbf{r},t)$), it is also possible to obtain equation (3) for the electric (magnetic) flux density if we assume a slowly-varying spatial and temporal evolution in μ_r (ε_r). The slowly-varying temporal evolution in a constitutive parameter, e.g. μ_r, requires to assume $|\delta_t\mu_\mathrm{r}| \ll |\mu_\mathrm{r}(t)|$ in $\delta t \sim 2\pi/\omega$, with $\delta_t\mu_\mathrm{r} := \mu_\mathrm{r}(t+\delta t) - \mu_\mathrm{r}(t)$. In a similar way, the slowly-varying spatial evolution requires to assume $|\delta_\mathbf{r}\mu_\mathrm{r}| \ll |\mu_\mathrm{r}(\mathbf{r},t)|$ in $|\delta\mathbf{r}| \sim \lambda$, with $\delta_\mathbf{r}\mu_\mathrm{r} := \mu_\mathrm{r}(\mathbf{r}+\delta\mathbf{r},t) - \mu_\mathrm{r}(\mathbf{r},t)$ and λ being the maximum wavelength of the problem.

In the isotropic homogeneous case ($n(\mathbf{r},t) = n_\mathrm{T}(t)$), Supplementary Equation 7 reduces to the familiar form of the wave equation:

$$\left(\triangle - \frac{n_\mathrm{T}^2(t)}{c_0^2}\frac{\partial^2}{\partial t^2}\right)\mathbf{D}(\mathbf{r},t) = \mathbf{0}. \tag{8}$$

As a final remark, although it is out of the scope of this work, it can also be demonstrated that equation (3) also follows in the case of nonlocal media by applying separation of variables in the electromagnetic fields, provided that the nonlocal and time-varying nature of the constitutive parameters can be decoupled.

Boundary conditions and polarisation dependence

The natural boundary conditions of the temporal Helmholtz equation (3) arise by noticing that ψ must be twice differentiable, i.e., such conditions are the continuity of ψ and $\dot{\psi}$. Along this line, note that the scattering properties and the eigenvalue degeneracy between both T-SUSY systems are preserved *if and only if* their temporal evolution fulfils these boundary conditions. Furthermore, it should be noted that these temporal boundary conditions are also decoupled from the spatial part of the problem, governed by the wave equation:

$$\nabla\times\nabla\times\left(\boldsymbol{\varepsilon}_\mathrm{S}^{-1}(\mathbf{r})\,\boldsymbol{\Phi}(\mathbf{r})\right) - \frac{\omega^2}{c_0^2}n_-^2\,\boldsymbol{\Phi}(\mathbf{r}) = \mathbf{0}. \tag{9}$$

Under the previous assumptions, the temporal and spatial wave equations (and their corresponding boundary conditions) are completely uncoupled. Since the response of the medium as a function of the polarisation state is fully determined by the spatial wave equation, this implies that the temporal wave equation has no influence on the polarisation dependence of the optical system. Therefore, if a system is polarisation-independent (or dependent), so will be its T-SUSY counterpart.

As an illustrative example, consider an ideal all-dielectric time-invariant polariser, which completely reflects one polarisation and completely transmits the orthogonal one. This device can be modelled by a time-invariant anisotropic heterogeneous medium characterised by an electric permittivity tensor $\boldsymbol{\varepsilon}_\mathrm{r1}(\mathbf{r},t) = n_\mathrm{T1}^2(t)\,\boldsymbol{\varepsilon}_\mathrm{S}(\mathbf{r}) = n_-^2\,\boldsymbol{\varepsilon}_\mathrm{S}(\mathbf{r})$. Now consider a reflectionless T-SUSY temporal refractive index partner of $\boldsymbol{\varepsilon}_\mathrm{r1}(\mathbf{r},t)$ given by $\boldsymbol{\varepsilon}_\mathrm{r2}(\mathbf{r},t) = n_\mathrm{T2}^2(t)\,\boldsymbol{\varepsilon}_\mathrm{S}(\mathbf{r})$ at a design frequency $\omega = \omega_0$. For simplicity, assume that $n_\mathrm{T2}(t\to-\infty) = n_-$ (note that this implies that $n_\mathrm{T1}(t\to\infty) = n_\mathrm{T2}(t\to\infty) \equiv n_+$ with $n_+ = n_-$, see Supplementary Equation 20). Hence, in line with Supplementary Equation 4, the electric flux density in each device can be expressed as:

$$\mathbf{D}^{(1,2)}(\mathbf{r},t) = \psi^{(1,2)}(t)\,\boldsymbol{\Phi}(\mathbf{r}), \tag{10}$$

where $\boldsymbol{\Phi}(\mathbf{r})$ is the solution to Supplementary Equation 9 and $\psi^{(1,2)}(t)$ is the solution to equation (3).

Now, assume that the original time-invariant polariser occupies the region $0 \le z \le L$ and that it completely reflects (transmits) the x-polarised (y-polarised) component of a z-propagating wave with an angular frequency ω_0. For this device, the solution to equation (3) is just $\psi^{(1)}(t) = \exp(i\omega_0 t)$ so, according to our assumption and to Supplementary Equation 10 we have:

$$\mathbf{D}^{(1)}(\mathbf{r},t) = \psi^{(1)}(t)\,\boldsymbol{\Phi}(\mathbf{r}) = \exp(i\omega_0 t)\begin{cases} A\cos(kz+\varphi_\mathrm{R}/2)\,\hat{\mathbf{x}} + B\exp(-ikz)\,\hat{\mathbf{y}}, & z<0 \\ B\exp(-i(kz+\varphi_\mathrm{T}))\,\hat{\mathbf{y}}, & z>L \end{cases}, \tag{11}$$

where A and B are complex constants, and where φ_{R} and φ_{T} are real constants encoding the phase shift introduced by the polariser for the reflected and transmitted waves, respectively. Along this line, it should be noted that the cosine term in Supplementary Equation 11 emerges from the linear combination of the incident and reflected x-polarised waves:

$$\begin{aligned} A'\left[\exp\left(-ikz\right)+\exp\left(i\varphi_{\mathrm{R}}\right)\exp\left(ikz\right)\right] &= A'\exp\left(i\frac{\varphi_{\mathrm{R}}}{2}\right)\left[\exp\left(-i\left(kz+\frac{\varphi_{\mathrm{R}}}{2}\right)\right)+\exp\left(i\left(kz+\frac{\varphi_{\mathrm{R}}}{2}\right)\right)\right] \\ &= 2A'\exp\left(i\frac{\varphi_{\mathrm{R}}}{2}\right)\cos\left(kz+\frac{\varphi_{\mathrm{R}}}{2}\right)\equiv A\cos\left(kz+\frac{\varphi_{\mathrm{R}}}{2}\right). \end{aligned} \tag{12}$$

On the other hand, since $n_{\mathrm{T2}}(t)$ is reflectionless and $n_- = n_+$, we will have:

$$\psi^{(2)}(t) \underset{t\to\infty}{\sim} \psi_+^{(2)}(t) = T_2 \exp\left(i\omega_0 t\right), \tag{13}$$

with $|T_2| = 1$ and the symbol $\sim$ representing an equivalence relation (for a time-invariant medium as the initial polariser, the temporal transmission coefficient is $T_1 = 1$. Additionally, $|T_2| = |T_1|$ from Supplementary Equation 27). Hence, after the time modulation (at $t \to \infty$), the complete solution for $\mathbf{D}$ in the T-SUSY polariser will be:

$$\mathbf{D}^{(2)}(\mathbf{r},t) \underset{t\to\infty}{\sim} \psi_+^{(2)}(t)\,\mathbf{\Phi}(\mathbf{r}) = T_2\exp\left(i\omega_0 t\right)\begin{cases} A\cos\left(kz+\varphi_{\mathrm{R}}/2\right)\hat{\mathbf{x}} + B\exp\left(-ikz\right)\hat{\mathbf{y}}, & z<0 \\ B\exp\left(-i\left(kz+\varphi_{\mathrm{T}}\right)\right)\hat{\mathbf{y}}, & z>L \end{cases}. \tag{14}$$

That is, the fields are exactly the same as in the time-independent initial polariser characterised by $\boldsymbol{\varepsilon}_{\mathrm{r1}}(\mathbf{r},t)$, except for a possible global phase shift introduced by T_2, which equally multiplies the incident and reflected waves (i.e., those at $z<0$) and the transmitted wave (that at $z>L$). Therefore, the reflectionless temporal index n_{T2} preserves the spatial scattering properties of $\boldsymbol{\varepsilon}_{\mathrm{r1}}(\mathbf{r},t)$ (relation between these waves) for both polarisations, having no influence on the polarisation dependence of the optical system, in this case the ability of completely reflecting one polarisation and fully transmitting the orthogonal one.

Supplementary Note 2: Temporal scattering – T-SUSY theory

In all the aforementioned media, the temporal evolution of $\mathbf{D}$ ($\mathbf{B}$), encoded by the ψ function, obeys equation (3) of the main text, reproduced here for clarity:

$$\left(\frac{\mathrm{d}^2}{\mathrm{d}t^2} + \omega^2 N^2\left(t\right)\right)\psi\left(t\right) = 0, \tag{15}$$

where $N^2\left(t\right) := n_-^2/n_{\mathrm{T}}^2\left(t\right)$, $n_- := n_{\mathrm{T}}\left(t \to -\infty\right)$, and ω is the angular frequency of ψ at $t \to -\infty$. Supplementary Equation 15 matches the 1D time-independent Schrödinger equation (equation (1) of the paper) taking $\alpha = 1$, performing the relabelling $x \to t$, and identifying:

$$\Omega - V\left(t\right) \equiv \omega^2 N^2\left(t\right). \tag{16}$$

In this way, assuming Ω (the eigenvalue in equation (1)) as a degree of freedom of the problem, we will be able to use the algebraic transformations of 1D SUSYQM in the time domain, which will give rise to time-varying refractive index profiles $n_{\mathrm{T}1,2}\left(t\right)$ with similar scattering properties at ω, provided that we use real $V_{1,2}$ potentials [1, 2].

A spatial or temporal scattering problem is well-defined when we can observe an incident wave and at least one reflected or transmitted wave. In the temporal scattering case, the incident wave is always defined at $t \to -\infty$ and the reflected and transmitted waves are found at $t \to \infty$. Thus, in order to have a well-defined *temporal* scattering problem in both superpartners, defined on the full line ($t \in \overline{\mathbb{R}}$), we require that:

$$\psi^{(1,2)}\left(t \to \pm\infty\right) \neq 0. \tag{17}$$

A sufficient condition to satisfy Supplementary Equation 17 is to consider $V_{1,2}\left(t \to \pm\infty\right) < \infty$, which is fulfilled by assuming: (*i*) $W_\pm := W\left(t \to \pm\infty\right)$ exists and is finite, and (*ii*) W' is uniformly continuous on the full line. Thus, from Barbalat's lemma [3] and Riccati's equation ($V_{1,2} = W^2 \mp W'$), we can infer that $W'_\pm = 0$ and $V_{1,\pm} = V_{2,\pm} = W_\pm^2 < \infty$. To summarise, we will have a well-defined scattering problem in both superpartners with:

$$\left.\begin{array}{c} |W_\pm| < \infty \\ W' \text{ unif. cont.} \end{array}\right\} \Rightarrow V_\pm \equiv V_{1,\pm} = V_{2,\pm} = W_\pm^2 < \infty \Rightarrow \psi^{(1,2)}\left(t \to \pm\infty\right) \neq 0. \tag{18}$$

Hence, combining Supplementary Equations 16 and 18, keeping in mind that both superpartners share the same eigenvalue Ω [1], we infer that:

$$N_1^2\left(t \to \pm\infty\right) = N_2^2\left(t \to \pm\infty\right) \equiv N_\pm^2, \tag{19}$$

with $N_- = 1$ by definition. From the above equation, the following remarks are in order:

- In spite of the fact that $N_{1,+}^2 = N_{2,+}^2$, note that $n_{1,+}^2 \neq n_{2,+}^2$ when $n_{1,-}^2 \neq n_{2,-}^2$. This can be observed when the SUSY systems are implemented over different background materials.
- If we assume positive-real constitutive parameters, $N_{1,\pm} = N_{2,\pm} \equiv N_\pm$, and then:

$$\frac{n_{1,-}}{n_{1,+}} = \frac{n_{2,-}}{n_{2,+}}. \tag{20}$$

In order to connect the temporal scattering problem of both superpartners, consider a plane wave in each system at $t \to -\infty$ with an angular frequency ω. In this way, the asymptotic behaviour of $\psi^{(1,2)}$ at $t \to -\infty$ is equivalent to:

$$\psi^{(1,2)}\left(t\right) \underset{t \to -\infty}{\sim} \psi_-^{(1,2)}\left(t\right) = \exp\left(iN_-\omega t\right). \tag{21}$$

Next, after the interaction with the refractive index variations $n_{\mathrm{T}1,2}(t)$, the asymptotic behaviour of $\psi^{(1,2)}$ at $t \to \infty$ will be found to be equivalent to:

$$\psi^{(1,2)}(t) \underset{t\to\infty}{\sim} \psi_{+}^{(1,2)}(t) = R_{1,2} \exp(-iN_{+}\omega t) + T_{1,2} \exp(iN_{+}\omega t), \tag{22}$$

$R_{1,2}$ and $T_{1,2}$ being respectively the reflection and transmission coefficients (which are equal to the complex amplitudes of the reflected and transmitted waves when the amplitude of the incident wave is set to 1), and $\omega N_{\pm} = \sqrt{\Omega - W_{\pm}^{2}}$. In such a scenario, using the SUSY relation $\psi^{(1)} = \xi \widehat{\mathrm{A}}^{+} \psi^{(2)}$ [1], where $\xi \in \mathbb{C}$, $\widehat{\mathrm{A}}^{\pm} := \mp \mathrm{d}/\mathrm{d}t + W(t)$ are the SUSY operators and W is the superpotential (a real-valued function in our case), we can relate the asymptotic behaviours as:

$$\psi_{\pm}^{(1)}(t) = \xi \left(-\frac{\mathrm{d}}{\mathrm{d}t} + W_{\pm}\right) \psi_{\pm}^{(2)}(t). \tag{23}$$

Thus, we have at $t \to -\infty$:

$$\exp(i\omega t) = \xi(-i\omega + W_{-}) \exp(i\omega t), \tag{24}$$

and at $t \to \infty$:

$$\begin{aligned} R_1 \exp(-iN_{+}\omega t) + T_1 \exp(iN_{+}\omega t) = {} & \xi R_2 (iN_{+}\omega + W_{+}) \exp(-iN_{+}\omega t) \\ & + \xi T_2 (-iN_{+}\omega + W_{+}) \exp(iN_{+}\omega t). \end{aligned} \tag{25}$$

Finally, equating terms with the same exponent in Supplementary Equations 24 and 25, we find:

$$\frac{R_1}{R_2} = \frac{W_{+} + iN_{+}\omega}{W_{-} - i\omega}; \quad \frac{T_1}{T_2} = \frac{W_{+} - iN_{+}\omega}{W_{-} - i\omega}. \tag{26}$$

Let us take a closer look at the above equations. In particular, it should be remarked that:

1. The SUSY refractive index profiles have identical intensity scattering behaviour:

   $$|R_1|^2 = |R_2|^2; \quad |T_1|^2 = |T_2|^2, \tag{27}$$

   as a direct consequence of the fact that both systems share the same eigenvalue $\Omega = \omega^2 N_{\pm}^2 + W_{\pm}^2$.

2. Although we consider complex-valued wave functions, the superpotential and the potentials must be real-valued functions to guarantee Supplementary Equation 27. Otherwise, the intensity scattering behaviour could be different in each system [2].

3. Supplementary Equation 26 does not depend on $n_{1,2,-}$. Consequently, we can engineer time-varying refractive index profiles exhibiting the same intensity scattering behaviour using the same ($n_{1,-} = n_{2,-}$) or different ($n_{1,-} \neq n_{2,-}$) background materials.

4. Bearing in mind that $N_{-} = 1$, we find that $\Omega = \omega^2 + W_{-}^2$. Therefore, we infer that $\Omega \geq \omega^2$.

5. The use of negative frequencies in Supplementary Equation 25 allows us, not only to describe adequately the temporal scattering problem (as discussed in the main text), but also to decouple the ratios R_1/R_2 and T_1/T_2. If we used only positive frequencies and negative wave numbers, we would find a scattering relation of the form:

   $$\frac{R_1 + T_1}{R_2 + T_2} = \frac{W_{+} - iN_{+}\omega}{W_{-} - i\omega}, \tag{28}$$

   that is, without the possibility of decoupling the reflected and transmitted amplitudes.

6. The ratios of the scattering coefficients $\mathcal{R}_i$ and $\mathcal{T}_i$ of the electric (magnetic) field strength are analogous to those in Supplementary Equation 26, since $\mathcal{R}_i = N_+^2 R_i$ and $\mathcal{T}_i = N_+^2 T_i$. Hence, we find that $\mathcal{R}_1/\mathcal{R}_2 = R_1/R_2$ and $\mathcal{T}_1/\mathcal{T}_2 = T_1/T_2$.

7. Note that $|W_+| = |W_-| \Leftrightarrow N_+ = N_- = 1 \Leftrightarrow n_{i,+} = n_{i,-}$. Such a situation takes place:
   - If $W_+ = -W_-$, in which case SUSY is said to be unbroken. Here, we observe that $R_1 = -R_2$. The reflected wave has an extra phase shift of π rad in the SUSY system.
   - If $W_+ = W_-$, in which case SUSY is said to be broken. Here, we find that $T_1 = T_2$.

8. In a SUSY refractive index chain $\{n_{\mathrm{T}s}(t)\}_{s=1}^{m}$ where the scattering problem is well-defined, the value of the potentials $\{V_s\}_{s=1}^{m}$ and superpotentials $\{W_s\}_{s=1}^{m-1}$ at $t \to \pm\infty$ is the same. That is:
$$V_{1,\pm} = V_{2,\pm} = \ldots = V_{m,\pm} = W_{1,\pm}^2, \tag{29}$$
and $W_{1,\pm} = W_{2,\pm} = \ldots = W_{m-1,\pm} \equiv W_\pm$. Consequently, the scattering coefficients of $n_{\mathrm{T}m}$ and $n_{\mathrm{T}1}$ are related by the following expressions:
$$\frac{R_1}{R_m} = \left(\frac{W_+ + iN_+\omega}{W_- - i\omega}\right)^{m-1}; \quad \frac{T_1}{T_m} = \left(\frac{W_+ - iN_+\omega}{W_- - i\omega}\right)^{m-1}. \tag{30}$$

On the other hand, given a refractive index $n_{\mathrm{T}1}(t) = n_{1,-}/N_1(t)$, its SUSY profile $n_{\mathrm{T}2}(t) = n_{2,-}/N_2(t)$ can be directly found by combining Supplementary Equation 16 and Riccati's equation ($V_{1,2} = W^2 \mp W'$):
$$n_{\mathrm{T}2}(t) = \frac{n_{2,-}}{\sqrt{\frac{n_{1,-}^2}{n_{\mathrm{T}1}^2(t)} - \frac{2}{\omega^2}W'(t)}}, \tag{31}$$
where Ω and $n_{2,-}$ are system parameters ($\equiv$degrees of freedom) of the problem. As mentioned above, $n_{2,-}$ allows us to change the background material of $n_{\mathrm{T}2}(t)$ while preserving the same intensity scattering properties as the original modulation $n_{\mathrm{T}1}(t)$, and the Ω parameter can be employed to tailor different features of $n_{\mathrm{T}2}(t)$, such as its maximal excursion (see Supplementary Note 3).

Shape Invariant Potentials (SIP)

Shape invariant potentials (SIP) are of great interest in quantum mechanics to find new analytically solvable potentials [1]. In the framework of SUSY quantum mechanics, SIP allows us to: (*i*) calculate the spectrum of a given potential and its (unbroken or broken) SUSY Hamiltonian chain in a simple and elegant way, and (*ii*) analyse and design the scattering properties of a large number of potentials. In particular, we are interested in this second feature.

In general, we will say that two SUSY partner potentials $V_{1,2}$ are *shape invariant* if they obey the relation [1]:
$$V_2(t;\mathbf{a}_1) = V_1(t;\mathbf{a}_2) + M(\mathbf{a}_1), \tag{32}$$
where $(\mathbf{a}_1, \mathbf{a}_2) \in \mathbb{R}^p \times \mathbb{R}^p$ are a set of parameters related by a multivariate function $\mathbf{f} \in \mathcal{F}(\mathbb{R}^p, \mathbb{R}^p)$ of the form $\mathbf{a}_2 = \mathbf{f}(\mathbf{a}_1)$, and $M \in \mathcal{F}(\mathbb{R}^p, \mathbb{R})$. In our numerical examples (see next section), we use $p = 1$.

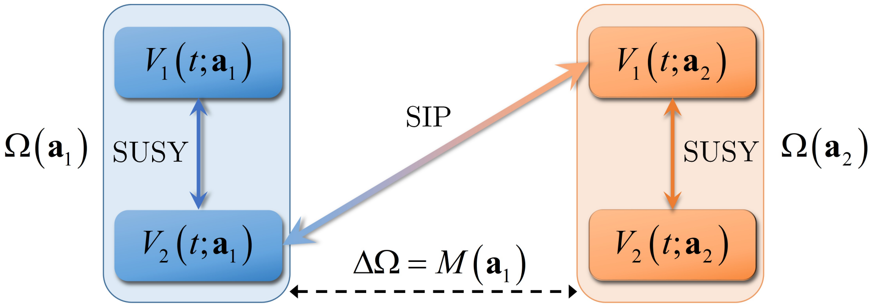

Supplementary Figure 1. Eigenvalue relation between SIP superpartners supporting continuous spectra.

From the above equation, we can infer the following properties of the temporal scattering problem, with the QM superpartners supporting a continuous spectrum:

- Supplementary Equation 32 establishes an eigenvalue relation between superpartners of the form (see Supplementary Figure 1):
$$\Omega\left(\mathbf{a}_1\right)=\Omega\left(\mathbf{a}_2\right)+M\left(\mathbf{a}_1\right), \tag{33}$$
where $\Omega\left(\mathbf{a}_i\right)\in I_i\subset\mathbb{R}$ and I_i is the eigenvalue spectrum of the SUSY partners $V_{1,2}\left(\mathbf{a}_i\right)$.

- Since $V_{i,\pm}\left(\mathbf{a}_j\right)=W_\pm^2\left(\mathbf{a}_j\right)\ \forall\left(i,j\right)\in\left\{1,2\right\}^2$, then:
$$W_\pm^2\left(\mathbf{a}_1\right)=W_\pm^2\left(\mathbf{a}_2\right)+M\left(\mathbf{a}_1\right). \tag{34}$$
Consequently, the superpotential depends on the SIP parameters at $t\to\pm\infty$.

- As demonstrated in [1], the wave functions are connected as:
$$\psi^{(2)}\left(t;\mathbf{a}_1\right)=\psi^{(1)}\left(t;\mathbf{a}_2\right). \tag{35}$$

- Combining Supplementary Equations 32 and 33 in $\Omega\left(\mathbf{a}_1\right)-V_2\left(t;\mathbf{a}_1\right)=\omega^2N_2^2\left(t;\mathbf{a}_1\right)$ we find that $N_2\left(t;\mathbf{a}_1\right)=N_1\left(t;\mathbf{a}_2\right)$, and therefore:
$$n_{\mathrm{T}2}\left(t;\mathbf{a}_1\right)=\frac{n_{2,-}\left(\mathbf{a}_1\right)}{n_{1,-}\left(\mathbf{a}_2\right)}n_{\mathrm{T}1}\left(t;\mathbf{a}_2\right), \tag{36}$$
with $n_{2,-}\left(\mathbf{a}_1\right)$ a degree of freedom of the problem.

- Interestingly, $N_\pm$ does not depend on the SIP parameters at $t\to\pm\infty$. Using Supplementary Equations 33 and 34:
$$\begin{aligned}\omega^2N_\pm^2\left(\mathbf{a}_1\right)&=\Omega\left(\mathbf{a}_1\right)-W_\pm^2\left(\mathbf{a}_1\right)=\Omega\left(\mathbf{a}_2\right)+M\left(\mathbf{a}_1\right)-W_\pm^2\left(\mathbf{a}_2\right)-M\left(\mathbf{a}_1\right)\\&=\Omega\left(\mathbf{a}_2\right)-W_\pm^2\left(\mathbf{a}_2\right)=\omega^2N_\pm^2\left(\mathbf{a}_2\right),\end{aligned} \tag{37}$$
we verify that $N_\pm\left(\mathbf{a}_1\right)=N_\pm\left(\mathbf{a}_2\right)$.

- From Supplementary Equation 35, we can infer that $R_2\left(\mathbf{a}_1\right)=R_1\left(\mathbf{a}_2\right)$ and $T_2\left(\mathbf{a}_1\right)=T_1\left(\mathbf{a}_2\right)$. As a result, Supplementary Equation 26 can be restated as:
$$\frac{R_1\left(\mathbf{a}_1\right)}{R_1\left(\mathbf{a}_2\right)}=\frac{W_+\left(\mathbf{a}_1\right)+iN_+\omega}{W_-\left(\mathbf{a}_1\right)-i\omega};\quad\frac{T_1\left(\mathbf{a}_1\right)}{T_1\left(\mathbf{a}_2\right)}=\frac{W_+\left(\mathbf{a}_1\right)-iN_+\omega}{W_-\left(\mathbf{a}_1\right)-i\omega}. \tag{38}$$

- In a SUSY refractive index chain with m shape invariant potentials in the continuum, Supplementary Equation 32 can be generalised to:
$$V_m\left(t;\mathbf{a}_1\right)=V_1\left(t;\mathbf{a}_m\right)+\sum_{i=1}^{m-1}M\left(\mathbf{a}_i\right), \tag{39}$$
with $\mathbf{a}_i=(\mathring{\mathbf{f}})^{i-1}\left(\mathbf{a}_1\right)$, e.g., $\mathbf{a}_3=(\mathring{\mathbf{f}})^2\left(\mathbf{a}_1\right)=\left(\mathbf{f}\circ\mathbf{f}\right)\left(\mathbf{a}_1\right)=\mathbf{f}(\mathbf{f}(\mathbf{a}_1))$. Hence, Supplementary Equations 33-36 become:
$$\Omega\left(\mathbf{a}_1\right)=\Omega\left(\mathbf{a}_m\right)+\sum_{i=1}^{m-1}M\left(\mathbf{a}_i\right); \tag{40}$$
$$W_\pm^2\left(\mathbf{a}_1\right)=W_\pm^2\left(\mathbf{a}_m\right)+\sum_{i=1}^{m-1}M\left(\mathbf{a}_i\right); \tag{41}$$
$$\psi^{(m)}\left(t;\mathbf{a}_1\right)=\psi^{(1)}\left(t;\mathbf{a}_m\right); \tag{42}$$
$$n_{\mathrm{T}m}\left(t;\mathbf{a}_1\right)=\frac{n_{m,-}\left(\mathbf{a}_1\right)}{n_{1,-}\left(\mathbf{a}_m\right)}n_{\mathrm{T}1}\left(t;\mathbf{a}_m\right). \tag{43}$$

Thus, $R_m(\mathbf{a}_1) = R_1(\mathbf{a}_m)$, $T_m(\mathbf{a}_1) = T_1(\mathbf{a}_m)$ and the scattering relations given by Supplementary Equation 30 can be recast as:

$$\frac{R_1(\mathbf{a}_1)}{R_1(\mathbf{a}_m)} = \left(\frac{W_+(\mathbf{a}_1) + iN_+\omega}{W_-(\mathbf{a}_1) - i\omega}\right)^{m-1}; \quad \frac{T_1(\mathbf{a}_1)}{T_1(\mathbf{a}_m)} = \left(\frac{W_+(\mathbf{a}_1) - iN_+\omega}{W_-(\mathbf{a}_1) - i\omega}\right)^{m-1}. \tag{44}$$

Isospectral Transformations

In the next lines, we will discuss the possibility of using SUSY transformations in the time domain to construct, from a given refractive index $n_{\mathrm{T}1}(t)$, an m-parameter family of isospectral refractive index profiles $\widetilde{n}_{\mathrm{T}1}(t;\eta_1,\ldots,\eta_m)$, that is, time-varying optical systems with exactly the same scattering properties in module and phase as the original one.

The *one-parameter* isospectral family $\widetilde{V}_1(x;\eta_1)$ of a given potential $V_1(x)$ can be calculated as indicated in Section 7.1 of [1]. In this vein, using our quantum-optical analogy (Supplementary Equation 16), we find the one-parameter isospectral family $\widetilde{n}_{\mathrm{T}1}(t;\eta_1)$ of the refractive index $n_{\mathrm{T}1}(t)$ as:

$$\widetilde{n}_{\mathrm{T}1}(t;\eta_1) = \frac{\widetilde{n}_{1,-}(\eta_1)}{\sqrt{\frac{n_{1,-}^2}{n_{\mathrm{T}1}^2(t)} + \frac{2}{\omega^2}\frac{\mathrm{d}^2}{\mathrm{d}t^2}\ln\left[\eta_1 + \int^t \exp\left(-2\int^\alpha W(\beta)\,\mathrm{d}\beta\right)\mathrm{d}\alpha\right]}}, \tag{45}$$

with η_1 and $\widetilde{n}_{1,-}(\eta_1)$ degrees of freedom of the problem. Interestingly, the above family has exactly the same scattering coefficients as the original modulation, provided that we use a nonsingular superpotential family, which is fulfilled by taking $\eta_1 > 0$. It is straightforward to prove this statement. Let us denote the scattering coefficients of $\widetilde{n}_{\mathrm{T}1}(t;\eta_1)$ as $R_1(\eta_1)$ and $T_1(\eta_1)$. The ratios $R_1(\eta_1)/R_2$ and $T_1(\eta_1)/T_2$ can be expressed in the same form as Supplementary Equation 26, but replacing $W_\pm$ by $\widetilde{W}_\pm(\eta_1)$ and $N_\pm$ by $\widetilde{N}_\pm(\eta_1)$, where $\widetilde{N}_\pm(\eta_1) := \widetilde{N}_1(t\to\pm\infty;\eta_1) = \widetilde{n}_{1,-}(\eta_1)/\widetilde{n}_{\mathrm{T}1}(t\to\pm\infty;\eta_1)$ and:

$$\begin{aligned}\widetilde{W}_\pm(\eta_1) :&= \widetilde{W}(t\to\pm\infty;\eta_1)\\ &= \lim_{t\to\pm\infty}\left\{W(t) + \frac{\mathrm{d}}{\mathrm{d}t}\ln\left[\eta_1 + \int^t \exp\left(-2\int^\alpha W(\beta)\,\mathrm{d}\beta\right)\mathrm{d}\alpha\right]\right\}.\end{aligned} \tag{46}$$

Concretely, $\widetilde{W}(t;\eta_1)$ is the family of superpotentials connecting $\widetilde{V}_1(t;\eta_1)$ and $V_2(t)$ via Riccati's equation. In order to preserve the scattering properties between both superpartners, the superpotential family must be nonsingular (sufficient condition but not necessary) [1]. To this end, we set $\eta_1 > 0$. Finally, taking into account that $\widetilde{W}_\pm(\eta_1) = W_\pm$ and $\widetilde{N}_\pm(\eta_1) = N_\pm$, we demonstrate that $R_1(\eta_1) = R_1$ and $T_1(\eta_1) = T_1$.

In addition, it is worth highlighting the possibility of combining the one-parameter isospectral transformation along with SIP. More specifically, given two potentials $V_i(t;\mathbf{a}_1)$ and $V_{i+1}(t;\mathbf{a}_1)$ of a SUSY chain calculated respectively with SIP from $V_1(t;\mathbf{a}_i)$ and $V_1(t;\mathbf{a}_{i+1})$ by using Supplementary Equation 39, we can obtain the superpotential $W_i(t;\mathbf{a}_1)$ from:

$$W_i(t;\mathbf{a}_1) = W_-(\mathbf{a}_1) + \frac{1}{2}\int_{-\infty}^t \left[V_1(\tau;\mathbf{a}_{i+1}) - V_1(\tau;\mathbf{a}_i) + M(\mathbf{a}_i)\right]\mathrm{d}\tau, \tag{47}$$

and later calculate the family $\widetilde{W}_i(t;\mathbf{a}_1,\eta_1)$ to construct the isospectral family $\widetilde{n}_{\mathrm{T}i}(t;\mathbf{a}_1,\eta_1)$ with the same scattering properties in module and phase as those of $n_{\mathrm{T}i}(t;\mathbf{a}_1)$. As seen, the temporal scattering properties of a large number of time-varying optical systems can be analysed and designed by combining both strategies.

On the other hand, the *multi-parameter* isospectral family $\widetilde{n}_{\mathrm{T}1}(t;\eta_1,\ldots,\eta_m)$ can be calculated from the multi-parameter Darboux procedure detailed in Section 7.2 of [1]. Here, we only describe the different steps of this procedure applied to time-varying optical systems:

1. We start from a given refractive index profile $n_{\mathrm{T}1}(t)$ associated with a QM potential $V_1(t \to x)$ via Supplementary Equation 16. This potential must support bound states and must satisfy the sufficient conditions detailed at the beginning of this section to guarantee that the temporal scattering problem is well-defined (see Supplementary Equation 18).

2. Next, we generate the family $\widetilde{V}_1(x; \eta_1, \ldots, \eta_m)$ from $V_1(x)$ by using the multi-parameter Darboux procedure.

3. Finally, performing the relabelling $\widetilde{V}_1(x \to t; \eta_1, \ldots, \eta_m)$ we obtain the sought family:

$$\widetilde{n}_{\mathrm{T}1}(t; \eta_1, \ldots, \eta_m) = \frac{\widetilde{n}_{1,-}(\eta_1, \ldots, \eta_m)}{\sqrt{1 + \frac{1}{\omega^2}\left[V_{1,-} - \widetilde{V}_1(t; \eta_1, \ldots, \eta_m)\right]}}, \tag{48}$$

   where $\{\eta_1, \ldots, \eta_m\} \subset \mathbb{R}^+$ and $\widetilde{n}_{1,-}(\eta_1, \ldots, \eta_m)$ are degrees of freedom in the above equation.

Supplementary Note 3: Temporal scattering – numerical examples

In this section, we include additional numerical examples of the temporal scattering problem in T-SUSY optical systems. Note that, as explained in the main text, T-SUSY index profiles are calculated at a frequency $\omega = \omega_0$.

Transparent hyperbolic secant modulation

In the main text, we analysed the SUSY refractive index profile $n_2(\mathbf{r}, t) = n_{\mathrm{T}2}(t)$ of a constant and, therefore, transparent refractive index $n_1(\mathbf{r}, t) = n_{1,-}$. In such a case, using Supplementary Equation 16, we infer that the original potential is:

$$V_1(t) = \Omega - \omega_0^2, \tag{49}$$

which leads to a superpotential of the form:

$$W(t) = -\sqrt{\Omega - \omega_0^2}\tanh\left(\sqrt{\Omega - \omega_0^2}\,t\right). \tag{50}$$

Finally, using Supplementary Equation 31, we find the T-SUSY refractive index:

$$n_2(\mathbf{r}, t) = n_{\mathrm{T}2}(t) = \frac{n_{2,-}}{\sqrt{1 + \frac{2}{\omega_0^2}\left(\Omega - \omega_0^2\right)\mathrm{sech}^2\left(\sqrt{\Omega - \omega_0^2}\,t\right)}}, \tag{51}$$

with $\Omega \geq \omega_0^2$, as discussed on page 6. In the following, we will first discuss the case $\Omega > \omega_0^2$, and later, we will analyse the case $\Omega = \omega_0^2$.

Considering $\Omega > \omega_0^2$, we observe two degrees of freedom in Supplementary Equation 51: $n_{2,-}$ and Ω. The former allows us to implement the above refractive index modulation over different background materials, and the latter can be employed to tailor its maximal excursion (Δn) and its temporal width (Δt) (the parameter Δn is defined as $\Delta n := n_{2,-} - \min\{n_{\mathrm{T}2}(t)\}$ and the parameter Δt is defined as the half-width at $1/(2e)$ maximum of the temporal profile $n_{2,-} - n_{\mathrm{T}2}(t)$). Supplementary Figure 2 shows the refractive index $n_{\mathrm{T}2}(t)$ for different values of the ratio Ω/ω_0^2. As seen, the higher the value of Ω/ω_0^2 is, the higher Δn and the lower Δt are. Along this line, it can be noted that the value of Ω/ω_0^2 also allows us to select the phase shifting performed by n_2. Specifically, bearing in mind that $R_1 = 0$ and $T_1 = 1$, we find from Supplementary Equation 26 that $R_2 = 0$ and:

$$T_2 = |T_2|\exp\left(i\Phi_{T_2}\right) = \exp\left[-i\left(\pi + 2\arctan\frac{1}{\sqrt{\Omega/\omega_0^2 - 1}}\right)\right]. \tag{52}$$

Supplementary Figure 3 compares Supplementary Equation 52 with the numerical results calculated by solving Supplementary Equation 8 with COMSOL taking $n_{2,-} = 2$, $\omega_0 = 38$ rad $\cdot$ s^{-1} and $c_0 = 1$ m $\cdot$ s^{-1} to guarantee a low computational time of the numerical simulations (the conclusions detailed below and in the next numerical examples are found to be valid for any value of ω_0 and c_0). We can note that the transmitted amplitude T_2 calculated with T-SUSY is in good agreement with the numerical results of the wave equation.

These graphics, along with Fig. 2 of the paper, could be of great interest to design and synthesise a perfect omnidirectional, polarisation-independent, transparent and reconfigurable phase shifter. From the selected value Ω/ω_0^2, we can directly estimate the performed phase shifting (Supplementary Figure 3), the required Δn and Δt (Supplementary Figure 2), and the spectral band of transparency (Fig. 2). Remarkably, these results can be implemented in all-dielectric, all-magnetic materials, or a combination of both. In the latter case, despite the fact that we must assume a slowly-varying temporal evolution in

one of the constitutive parameters (see Supplementary Note 1), n_{T2} may also present rapidly-varying temporal fluctuations. Moreover, note that these results are independent of the value of ω_0 and only depend on the ratio Ω/ω_0^2. Accordingly, they can be directly extrapolated to a different angular frequency.

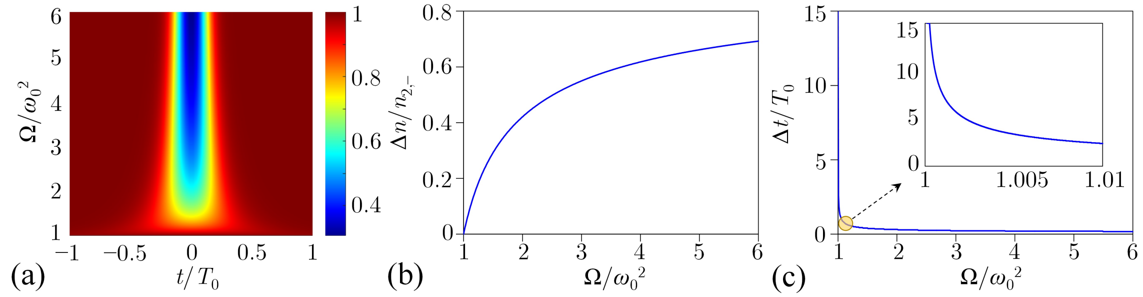

Supplementary Figure 2. Hyperbolic secant modulation Supplementary Equation 51. (a) Normalised refractive index profile $n_{\mathrm{T2}}(t)/n_{2,-}$, (b) normalised maximal excursion $\Delta n/n_{2,-}$, and (c) normalised temporal width $\Delta t/T_0$, with $T_0 = 2\pi/\omega_0$. All graphics have been normalised to guarantee the same results for any value of ω_0 and $n_{2,-}$.

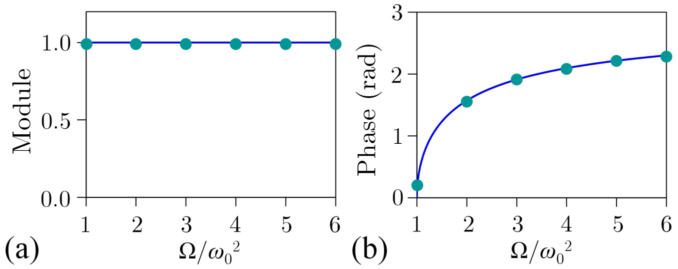

Supplementary Figure 3. Scattering coefficient T_2 given by Supplementary Equation 52 (blue line) and calculated numerically from Supplementary Equation 8 using COMSOL Multiphysics (dots). (a) Module and (b) phase as a function of the ratio Ω/ω_0^2.

In addition, as seen in Fig. 2e of the main text, Φ_{T_2} shows a flat frequency response. Consequently, in our phase shifter, the reconfigurable and frequency-independent response of the phase could be of extreme utility for wavelength-division multiplexing (WDM) transmissions of narrow-band signals to generate the same phase shifting in each WDM channel. In such a scenario, if we use electro-optic modulators with a reduced Δn excursion ($\Delta n \sim 10^{-3}$), we must operate in the range $\Omega/\omega_0^2 < 1.01$, where $\Phi_{T_2} < 1$ rad. In these circumstances, it would be of interest to us to concatenate a chain of non-reflecting hyperbolic secant modulations to increase the phase shifting induced by a single hyperbolic secant. Concretely, the hyperbolic secant chain (HSC) can be described as:

$$n_{\mathrm{HSC}}(\mathbf{r}, t) = \sum_{k=0}^{N_{\mathrm{HSC}}-1} n_{\mathrm{T2}}\left(t + \left(\frac{N_{\mathrm{HSC}}-1}{2} - k\right)\mathcal{T}_{\mathrm{HSC}}\right) - (N_{\mathrm{HSC}}-1)\, n_{2,-}, \tag{53}$$

where $\mathcal{T}_{\mathrm{HSC}}$ is the fundamental period of the chain and N_{HSC} is the number of fundamental periods (see Supplementary Figure 4a). In order to analyse the scattering behaviour of n_{HSC}, we numerically calculate the scattering coefficients R_{HSC} and T_{HSC} as a function of the ratio $\mathcal{T}_{\mathrm{HSC}}/T_{\mathrm{P}}$, where T_{P} is the full-width at $1/(2e)$ of the peak power of the incident pulse. Supplementary Figure 4 shows the numerical results of R_{HSC} and T_{HSC} taking: $n_{2,-} = 2$, $\Omega/\omega_0^2 = 1.01$ (Supplementary Figure 4b), $\Omega/\omega_0^2 = 6$ (Supplementary Figure 4c), $N_{\mathrm{HSC}} \in \{2, 3, 4\}$, $\mathcal{T}_{\mathrm{HSC}}/T_{\mathrm{P}} \in (0, 2]$, and $T_{\mathrm{P}} = 1$ s. We can observe that the module and phase of R_{HSC} and T_{HSC} have a flat behaviour as a function of $\mathcal{T}_{\mathrm{HSC}}/T_{\mathrm{P}}$, even if $\mathcal{T}_{\mathrm{HSC}} < T_{\mathrm{P}}$. Hence, as expected, we can use an HSC to increase the phase shifting of the original hyperbolic secant ($\Phi_{T_{\mathrm{HSC}}}(N_{\mathrm{HSC}}) = N_{\mathrm{HSC}}\Phi_{T_2}$) maintaining its non-reflecting nature.

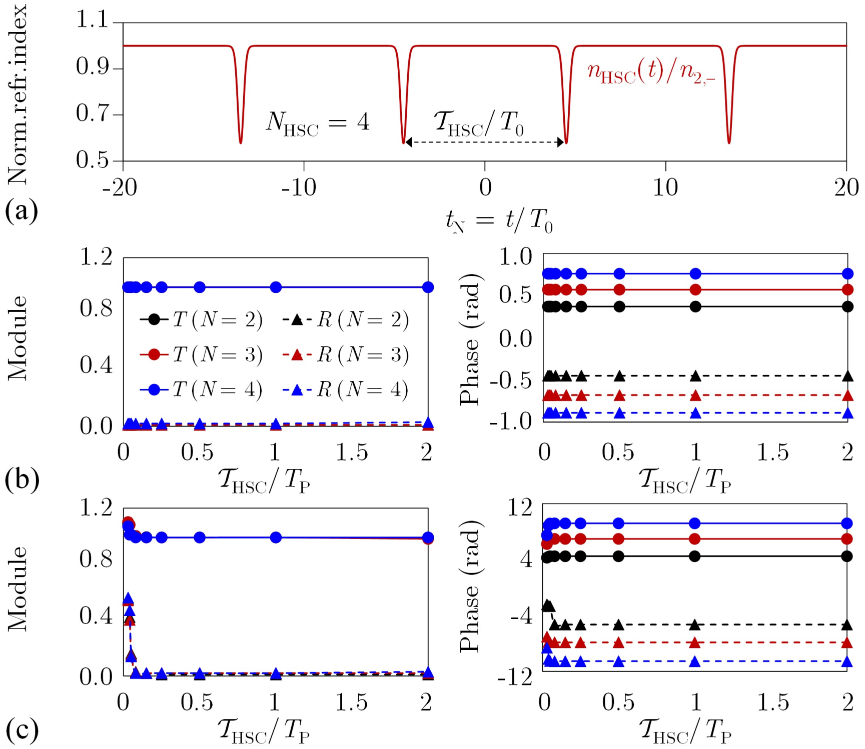

Supplementary Figure 4. Chain of non-reflecting hyperbolic secant refractive index profiles. (a) Normalised refractive index profile $n_{\mathrm{HSC}}(t)/n_{2,-}$. (b,c) Scattering coefficients T_{HSC} and R_{HSC} calculated numerically as a function of the ratio $\mathcal{T}_{\mathrm{HSC}}/T_{\mathrm{P}}$ for the case $n_{2,-} = 2$ and: (b) $\Omega/\omega_0^2 = 1.01$ and (c) $\Omega/\omega_0^2 = 6$. The number of fundamental periods N_{HSC} of the chain ranges from 2 to 4. The legend of (b) also applies to (c). We omit the subscripts of T_{HSC}, R_{HSC} and N_{HSC} in the legend due to space constraints.

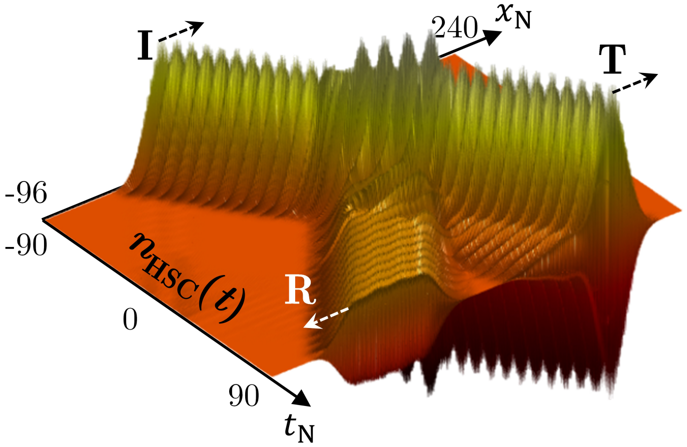

Supplementary Figure 5. Pulse propagation evolution through the time-varying medium described by $n_{\mathrm{HSC}}(t)$. Reflected (R) and transmitted (T) optical pulses generated from the interaction of an incident (I) optical pulse with an hyperbolic secant chain operating at $\omega = 1.4\omega_0$. The transmitted pulse has a higher peak power than the incident pulse because the energy is not conserved in a time-varying system ($\mathrm{I} \neq \mathrm{R} + \mathrm{T}$). The temporal axis is normalised as $t_{\mathrm{N}} = t/T_0$ with $T_0 = 2\pi/\omega_0$. The x-axis is normalised as $x_{\mathrm{N}} = x/\lambda$, with $\lambda = \lambda_0/n_{2,-}$ and $\lambda_0 = 2\pi c_0/\omega_0$.

On the other hand, the reflecting behaviour of $n_{\mathrm{T2}}(t)$ at $\omega \neq \omega_0$ and the nonlinear nature of $\Phi_{T_2}(\omega)$ (see Fig. 2e of the main text) can be exploited to implement pulse shaping operations in reflection and transmission. Specifically, in reflection, we can build a flat-top optical pulse using the HSC. The chain generates N_{HSC} reflected pulses of temporal width T_{P} and separated $\mathcal{T}_{\mathrm{HSC}}$ in time. Hence, selecting $T_{\mathrm{P}} \sim \mathcal{T}_{\mathrm{HSC}}$, we will obtain a flat-top optical pulse emerging from the superposition of all the reflected pulses. Supplementary Figure 5 illustrates this basic idea. We build an HSC with $n_{2,-} = 2$, $\Omega/\omega_0^2 = 6$, $\omega = 1.4\omega_0$, $\mathcal{T}_{\mathrm{HSC}}/T_{\mathrm{P}} = 1.2$, $T_{\mathrm{P}} = 1\,\mathrm{s}$ and $N_{\mathrm{HSC}} = 4$. As seen, a flat-top optical pulse can be observed in reflection at the end of the chain. Moreover, the tail of the transmitted pulse has been distorted due to the nonlinear nature of $\Phi_{T_2}(\omega)$ (the nonlinear frequency dependence of the phase $\Phi_{T_2}(\omega)$ implies that each spectral component of $\mathbf{D}(\mathbf{r},t)$ is phase shifted by a different quantity. Hence, the envelope of an incident wide-band pulse will be distorted in transmission). This can be further investigated in future works to generate transmitted optical pulses with an exotic shape, e.g., for optical wavelet transforms, coherent laser control of physicochemical processes, or spectrally selective nonlinear microscopy among other application areas [4–6]. On the contrary, the pulse distortion induced by the nonlinear frequency response of $\Phi_{T_2}(\omega)$ can be reduced if we operate with narrow-band incident pulses.

So far, we have extensively evaluated the SUSY refractive index of $n_1(\mathbf{r},t) = n_{1,-}$ by considering $\Omega > \omega_0^2$. Now, we focus our attention on the case $\Omega = \omega_0^2$. In such circumstances, $V_1(t) = 0$, $W(t) = 1/(C-t)$ and:

$$n_2(\mathbf{r},t) = n_{\mathrm{T2}}(t) = \frac{n_{2,-}}{\sqrt{1 - \frac{2}{\omega_0^2 (C-t)^2}}}, \tag{54}$$

where C is an integration constant arising from Riccati's equation. Supplementary Figure 6 illustrates the SUSY refractive index profile given by the above equation. It is fundamental to note that now we have a singular superpotential that may break the degeneracy of the eigenvalue spectra between both superpartners ($\Omega^{(1)} \neq \Omega^{(2)}$). More precisely, note that $\mathbf{D}^{(2)} \propto (\partial_t + W(t))\,\mathbf{D}^{(1)}$ is not a continuous function at $t = C$. Therefore, $\mathbf{D}^{(2)}$ cannot be a solution of Maxwell's equations. The boundary conditions of the temporal wave equation (equation (3) of the paper) are not satisfied ($\mathbf{D}^{(2)}$ and $\partial_t \mathbf{D}^{(2)}$ must be necessarily continuous functions in the temporal variable). As a result, we cannot guarantee the same intensity scattering properties for n_1 and n_2.

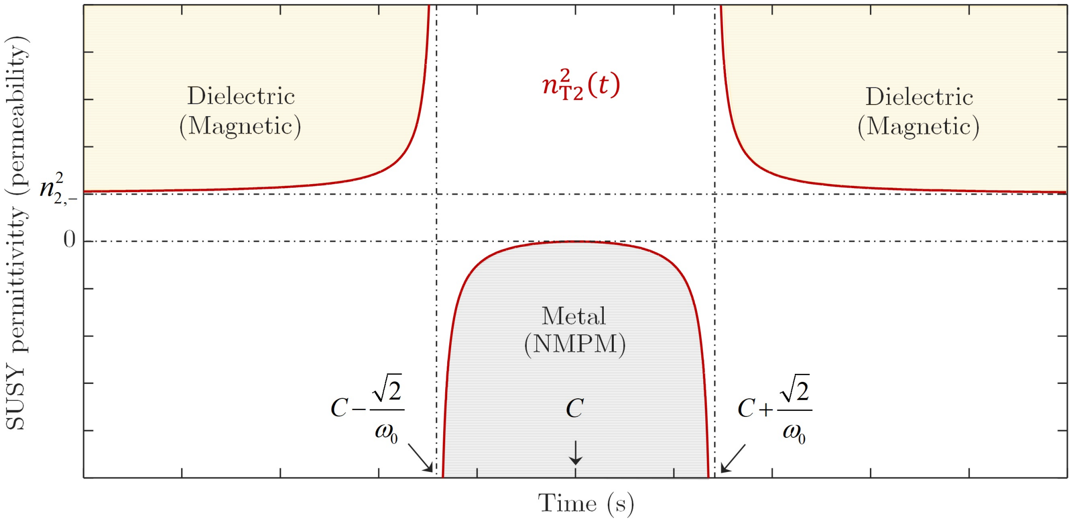

Supplementary Figure 6. T-SUSY permittivity (permeability) counterpart n_{T2}^2 of a constant permittivity (permeability) $n_{1,-}^2$ when $\Omega = \omega_0^2$. (NMPM: negative magnetic permeability material).

Hyperbolic Rosen-Morse II potential: general expressions

In the next two numerical examples, we will analyse the SUSY refractive index profiles arising from the shape invariant hyperbolic Rosen-Morse II (HRMII) potentials, described by the following equations:

$$W(t) = A\tanh(\alpha t) + B/A; \tag{55}$$

$$V_1(t) = A^2 + B^2/A^2 - A(A+\alpha)\,\mathrm{sech}^2(\alpha t) + 2B\tanh(\alpha t); \tag{56}$$

$$V_2(t) = A^2 + B^2/A^2 - A(A-\alpha)\,\mathrm{sech}^2(\alpha t) + 2B\tanh(\alpha t), \tag{57}$$

with A, B and α real parameters and $B < A^2$. From the above expressions, we can verify that the SIP condition (Supplementary Equation 32) is fulfilled with $a_1 \equiv A$, $a_2 = f(a_1) = a_1 - \alpha$ and $M(a_1) = a_1^2 - (a_1-\alpha)^2 + B^2[1/a_1^2 - 1/(a_1-\alpha)^2]$. Taking $\Omega(a_1) = \omega_0^2 + W_-^2 = \omega_0^2 + (B/a_1 - a_1)^2$, the corresponding refractive index profiles are:

$$n_{\mathrm{T1}}(t;a_1) = \frac{\omega_0 n_{1,-}(a_1)}{\sqrt{\omega_0^2 - 2B + a_1(a_1+\alpha)\,\mathrm{sech}^2(\alpha t) - 2B\tanh(\alpha t)}}; \tag{58}$$

$$n_{\mathrm{T2}}(t;a_1) = \frac{\omega_0 n_{2,-}(a_1)}{\sqrt{\omega_0^2 - 2B + a_1(a_1-\alpha)\,\mathrm{sech}^2(\alpha t) - 2B\tanh(\alpha t)}}. \tag{59}$$

Supplementary Equation 58 is equation (6) of the paper. We also observe that $|W_-| = |W_+|$ ($|W_-| \neq |W_+|$) if $B = 0$ ($B \neq 0$). Hence, we have the ability of constructing time-varying optical systems with $\omega_{i,-} = \omega_{i,+}$ ($\omega_{i,-} \neq \omega_{i,+}$). In the case $B \neq 0$, we should take into account that:

- The scattering coefficients of the electric (magnetic) flux density R_i and T_i are found to be different from the scattering coefficients of the electric (magnetic) field strength $\mathcal{R}_i$ and $\mathcal{T}_i$. More precisely, $\mathcal{R}_i = N_+^2 R_i$ and $\mathcal{T}_i = N_+^2 T_i$, with $N_+^2 = 1 - 4B/\omega_0^2 \in (0,1)$. Nonetheless, $\mathcal{R}_1/\mathcal{R}_2 = R_1/R_2$ and $\mathcal{T}_1/\mathcal{T}_2 = T_1/T_2$.
- From the previous point, we deduce that $0 < N_+ < 1$, $n_{i,-} < n_{i,+}$ and $B < \omega_0^2/4$.
- From the temporal version of Snell's law, we infer that the frequency of the transmitted signal is lower than that of the incident signal, with $\omega_{i,+} = N_+\omega_{i,-}$.

In both cases ($B = 0$ and $B \neq 0$), the scattering relations (Supplementary Equation 26) become:

$$\frac{R_1(a_1)}{R_2(a_1)} = \frac{R_1(a_1)}{R_1(a_2)} = \frac{a_1 + B/a_1 + i\sqrt{\omega_0^2 - 4B}}{-a_1 + B/a_1 - i\omega_0}; \tag{60}$$

$$\frac{T_1(a_1)}{T_2(a_1)} = \frac{T_1(a_1)}{T_1(a_2)} = \frac{a_1 + B/a_1 - i\sqrt{\omega_0^2 - 4B}}{-a_1 + B/a_1 - i\omega_0}. \tag{61}$$

Supplementary Figure 7 shows the above ratios as a function of ω_0 and a_1 setting $B = 0.4a_1^2$. As seen, the module is equal to 1 when $N_+ \in \mathbb{R}$ and the phase has a low dependence on ω_0. Furthermore, the lower the value of a_1 is, the lower the frequency dependence of the phase and the area where $N_+ \in \mathbb{C}$ are.

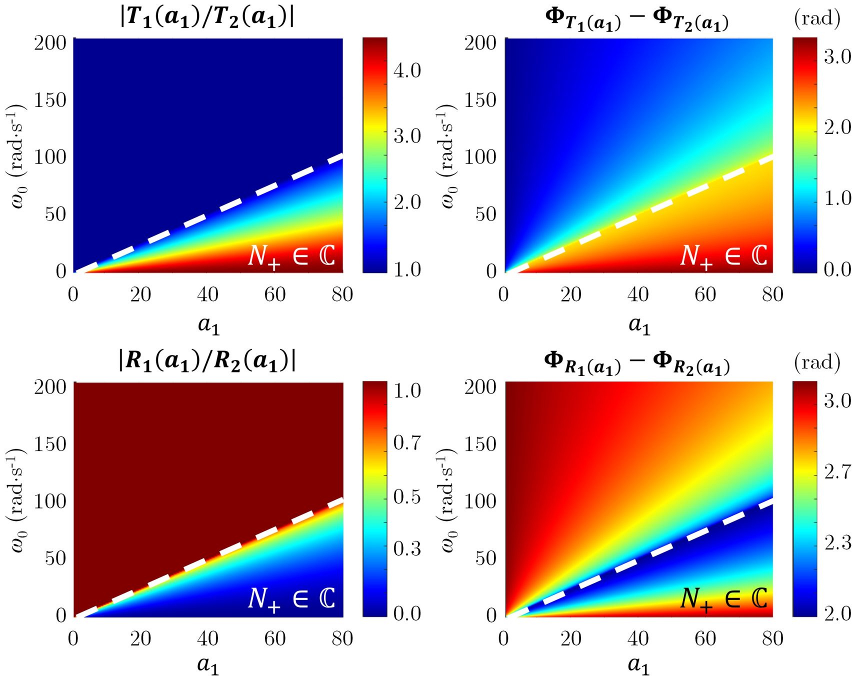

Supplementary Figure 7. Ratios of the scattering coefficients in the HRMII potential. The dashed white line separates the allowed ($N_+ \in \mathbb{R}$, top) and forbidden ($N_+ \in \mathbb{C}$, bottom) regions.

Hyperbolic Rosen-Morse II potential: case $B = 0$

The case $B = 0$ leads to reciprocal, omnidirectional, polarisation-independent and transparent phase shifters such as the hyperbolic secant profile of the first numerical example. Specifically, taking $\alpha = a_1$ in Supplementary Equation 58, we retrieve the aforementioned refractive index profile (Supplementary Equation 51). Nevertheless, setting $\alpha = 1$ and $a_1 = m \in \mathbb{Z}$, we will find new refractive index modulations with an extremely large transparent optical bandwidth. In such a scenario, Supplementary Equations 58 and 59 are reduced to:

$$n_{\mathrm{T}1}\left(t;m\right) = \frac{\omega_0 n_{1,-}\left(m\right)}{\sqrt{\omega_0^2 + m\left(m+1\right)\mathrm{sech}^2 t}}; \qquad (62)$$

$$n_{\mathrm{T}2}\left(t;m\right) = \frac{\omega_0 n_{2,-}\left(m\right)}{\sqrt{\omega_0^2 + m\left(m-1\right)\mathrm{sech}^2 t}}, \qquad (63)$$

obeying the SIP relation Supplementary Equation 36, which can be rewritten as:

$$n_{\mathrm{T}2}\left(t;m\right) = \frac{n_{2,-}\left(m\right)}{n_{1,-}\left(m-1\right)} n_{\mathrm{T}1}\left(t;m-1\right). \qquad (64)$$

From this recurrence relation, we can infer the transparent behaviour of these systems taking into account that they are SUSY-connected with the potential of the free particle ($m = 1$). Nevertheless, in contrast to the first numerical example (Supplementary Equation 51), we can note that the HRMII has the advantage of allowing independent design control over $\Delta n \sim n_{1,2,-}\left|1 - \omega_0/\sqrt{\omega_0^2 + m\left(m \pm 1\right)}\right|$ for a fixed $\Delta t \sim 10T_0$, enabling a technology-oriented adjustment of the index modulation contrast. Supplementary Figure 8a shows the normalised T-SUSY refractive index profiles for the case $m = 30$, and Supplementary Figure 8b depicts their scattering coefficients as a function of the frequency. As commented above, we can observe an extremely large spectral band of transparency.

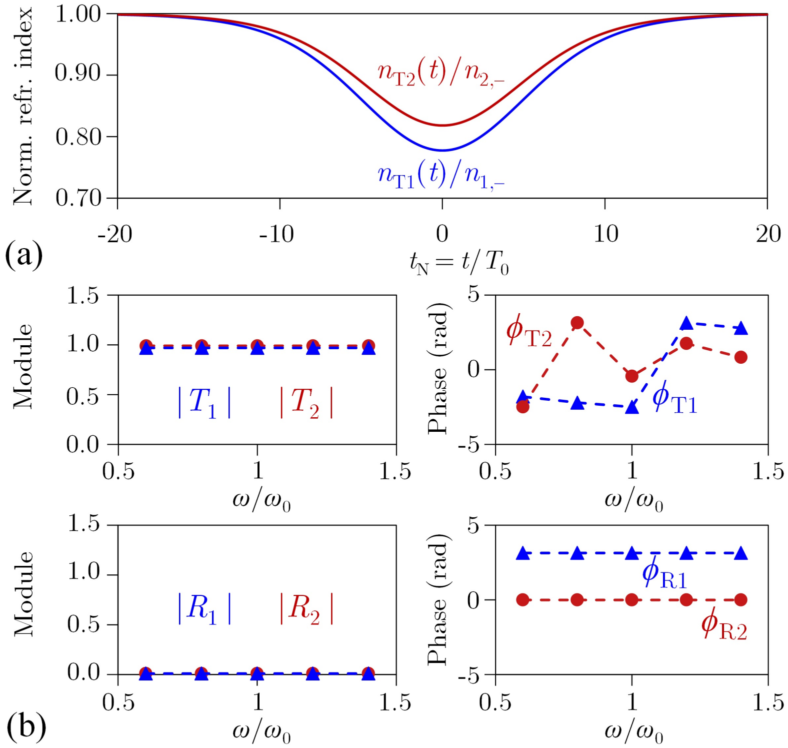

Supplementary Figure 8. (a) Normalised T-SUSY refractive index profiles given by Supplementary Equations 62 and 63 with $m = 30$. (b) Scattering coefficients calculated numerically as a function of the ratio ω/ω_0 for the case $n_{1,-} = n_{2,-} = 2$.

On the other hand, in this case we can report an analytic solution for $T_1(m)$, which can be calculated in the same way as the transmitted probability amplitude associated with the potential $V_1(x; m) = m^2 + \omega_0^2[1 - N_1^2(t \to x; m)]$ in quantum mechanics (see page 299 of [1]):

$$T_1(m) = \frac{\Gamma(-m - i\omega_0)\,\Gamma(m + 1 - i\omega_0)}{\Gamma(-i\omega_0)\,\Gamma(1 - i\omega_0)}, \tag{65}$$

where Γ is the Gamma function. Supplementary Figure 9 shows an excellent fitting between the theoretical and numerical transmission coefficient associated with $n_{T1}(t; m)$. Strikingly, Supplementary Equation 65 can be combined with Supplementary Equation 61 to solve straightforwardly the temporal scattering problem in this family of time-varying systems without using Maxwell's equations. Furthermore, we can select the desired phase of the transmitted wave via the m parameter.

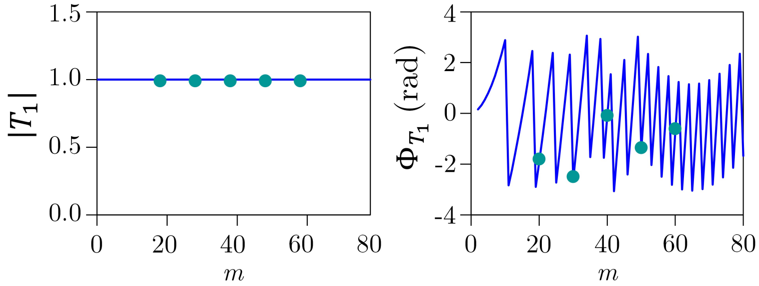

Supplementary Figure 9. Transmission coefficient associated with the refractive index profile given by Supplementary Equation 62 as a function of m. (Solid line: Supplementary Equation 65. Dots: numerical results).

Isospectral two-parameter family. Figure 3a of the paper depicts the two-parameter isospectral family $\widetilde{n}_{\mathrm{T1}}(t;\eta_1,\eta_2)$ of the HRMII index given by Supplementary Equation 62. Here, we detail how we have calculated $\widetilde{n}_{\mathrm{T1}}(t;\eta_1,\eta_2)$. Taking $\Omega=\omega_0^2$ in Supplementary Equation 16, the corresponding quantum potential is of the form (step 1 of the isospectral theory, see page 10):

$$V_1(t\to x)=\Omega-\omega_0^2\frac{n_{1,-}^2}{n_{\mathrm{T1}}^2(t\to x;m)}=-m(m+1)\,\mathrm{sech}^2 x. \tag{66}$$

In particular, V_1 holds m bound states and the scattering problem is well-defined ($V_{1,\pm}<\infty$). For the case $m=2$, the two-parameter isospectral family $\widetilde{V}_1(x;\eta_1,\eta_2)$ can be calculated from $V_1(x)$ by using the multi-parameter Darboux procedure (step 2):

$$\widetilde{V}_1(x;\eta_1,\eta_2)=-12\frac{3+4\cosh(2x-2\Lambda_2)+\cosh(4x-2\Lambda_1)}{\left[\cosh(3x-\Lambda_2-\Lambda_1)+3\cosh(x+\Lambda_2-\Lambda_1)\right]^2}, \tag{67}$$

with $\Lambda_{1,2}=-0.5\ln(1+1/\eta_{1,2})$ and $\eta_{1,2}>0$. Finally, performing the relabelling $\widetilde{V}_1(x\to t;\eta_1,\eta_2)$ and using Supplementary Equation 48, we obtain the two-parameter isospectral family of time-varying optical systems (step 3):

$$\widetilde{n}_{\mathrm{T1}}(t;\eta_1,\eta_2)=\frac{\widetilde{n}_{1,-}(\eta_1,\eta_2)}{\sqrt{1-\frac{1}{\omega_0^2}\widetilde{V}_1(t;\eta_1,\eta_2)}}. \tag{68}$$

Figure 3a of the main text shows the normalised refractive index profile of different optical systems of the family. We have numerically calculated the scattering coefficients of these refractive index profiles taking $\widetilde{n}_{1,-}(\eta_1,\eta_2)=n_{1,-}=2$ and we have found the same reflection and transmission coefficients in module and phase as those of the original modulation $n_{\mathrm{T1}}(t;m=2)$ at $\omega=\omega_0$: $R_1(\eta_1,\eta_2)=0$ and $T_1(\eta_1,\eta_2)=\exp(i0.23)$.

Hyperbolic Rosen-Morse II potential: case $B\neq 0$ (optical isolator)

The case $B\neq 0$ is of great interest to us given that it builds a bridge to design non-reciprocal optical systems using time-varying refractive index modulations, as commented above and in the main text. In particular, from the temporal SIP theory developed above, the value of n_{T6} employed for the optical isolator demonstrated in the paper can be obtained by replacing a_1 by $a_6=a_1-5\alpha$ in Supplementary Equation 58. The frequency down-conversion between the incident and transmitted waves, with angular frequencies $\omega_0\equiv\omega_{6,-}$ and $\omega_{6,+}$, respectively, can be calculated from the temporal version of Snell's law $\omega_{6,-}n_{6,-}=\omega_{6,+}n_{6,+}$. Hence, the ratio $\omega_{6,+}/\omega_{6,-}$ obeys the relation:

$$\frac{\omega_{6,+}}{\omega_{6,-}}=\frac{n_{6,-}}{n_{6,+}}=N_+=\sqrt{1-4B/\omega_0^2}. \tag{69}$$

Supplementary Figure 10 depicts the ratio $\omega_{6,+}/\omega_{6,-}$ for different values of ω_0 and B. The dashed line separates the allowed ($N_+\in\mathbb{R}$) and forbidden ($N_+\in\mathbb{C}$) areas. In our case, we operate at $\omega_{6,+}/\omega_{6,-}\simeq 0.7$ with $\omega_0\equiv\omega_{6,-}=38$ rad/s. If we are interested in synthesising the optical isolator of the paper for a different angular frequency, we must select an adequate value of B that preserves the same ratio in Supplementary Equation 69.

On the other hand, the ratios $R_1(a_1)/R_6(a_1)$ and $T_1(a_1)/T_6(a_1)$ can be calculated by combining Supplementary Equation 44 with Supplementary Equations 60 and 61. In particular, we find that:

$$\frac{T_1(a_1)}{T_6(a_1)}=\frac{T_1(a_1)}{T_1(a_6)}=\left(\frac{a_1+B/a_1-i\sqrt{\omega_0^2-4B}}{-a_1+B/a_1-i\omega_0}\right)^5=\exp(i2.55), \tag{70}$$

which is in good agreement with Fig. 3c of the main text at $\omega=\omega_0$. The ratio $R_1(a_1)/R_6(a_1)$ could not be numerically estimated because the non-reflecting behaviour of $n_{\mathrm{T1}}(t;a_1)$ and $n_{\mathrm{T6}}(t;a_1)$ has a flat frequency response in an extremely large optical bandwidth (see paper Fig. 3). We could not observe any reflected wave in the numerical simulation when propagating wide-band optical pulses through the above media.

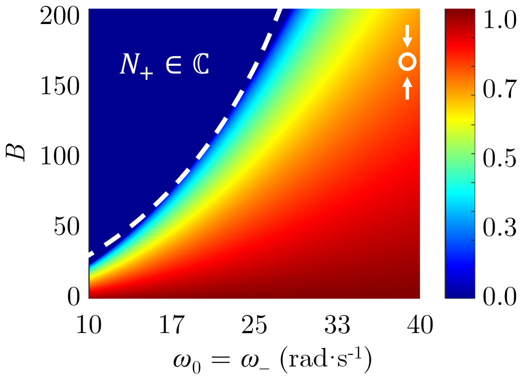

Supplementary Figure 10. Frequency down-conversion ratio $\omega_{6,+}/\omega_{6,-}$ as a function of ω_0 and B. The dashed white line separates the allowed ($N_+ \in \mathbb{R}$) and forbidden ($N_+ \in \mathbb{C}$) areas. The hollow circle inset in the colormap indicates the operation point of the optical isolator of the main text.

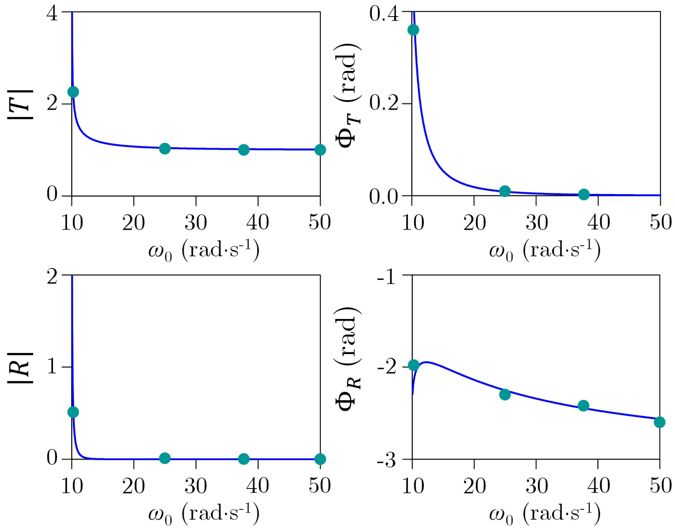

Supplementary Figure 11. Scattering coefficients of the hyperbolic step-index profile (Supplementary Equation 58 taking $\alpha = A = B^{1/2}$) as a function of ω_0 for the case $\alpha = 5$ and $n_{1,-} = 2$. (Solid line: Supplementary Equations 71 and 72. Dots: numerical results).

Interestingly, for the hyperbolic step-index profile with $\alpha = A = B^{1/2}$, we can find an analytic expression for the scattering coefficients (the mathematical derivation of these expressions is detailed below):

$$R = -\frac{1}{N_+}\frac{\Gamma\left(i\omega_0/\alpha\right)\Gamma\left(1 - i\omega_0 N_+/\alpha\right)}{\Gamma\left(i\omega_0\left(1 - N_+\right)/2\alpha\right)\Gamma\left(1 + i\omega_0\left(1 - N_+\right)/2\alpha\right)}; \tag{71}$$

$$T = \frac{1}{N_+}\frac{\Gamma\left(i\omega_0/\alpha\right)\Gamma\left(1 + i\omega_0 N_+/\alpha\right)}{\Gamma\left(i\omega_0\left(1 + N_+\right)/2\alpha\right)\Gamma\left(1 + i\omega_0\left(1 + N_+\right)/2\alpha\right)}. \tag{72}$$

Supplementary Figure 11 demonstrates a perfect fitting between the above expressions and the numerical results for a particular value of α. In this example, we can observe the non-reflecting behaviour of the hyperbolic step-index profile when $\omega_0 > 10$ rad/s. In conclusion, we can combine the theoretical tools provided by T-SUSY, SIP and Supplementary Equations 71 and 72 to design broadband polarisation-independent optical isolators.

***Derivation of Supplementary Equations* 71 *and* 72.** Following a similar strategy to that of [7], our first goal is to analyse the asymptotic behaviour of the general solution of the time-independent Schrödinger equation when considering the hyperbolic step potential:

$$V\left(x\right)=\frac{1}{2}V_0\left(1+\tanh\left(\frac{x}{2\widetilde{\alpha}}\right)\right), \tag{73}$$

with $V_0>0$ and $\widetilde{\alpha}>0$. The general solution to the equation $\psi''\left(x\right)+\left(E-V\left(x\right)\right)\psi\left(x\right)=0$ is given by equation 9 of [7], where $k=\sqrt{E}$ and $k'=\sqrt{E-V_0}$. Thus, using the asymptotic behaviour of the hypergeometric functions, we find that ($\psi\left(x\right)\underset{x\to-\infty}{\sim}\psi_-\left(x\right)$ and $\psi\left(x\right)\underset{x\to\infty}{\sim}\psi_+\left(x\right)$):

$$\begin{aligned}\psi_-\left(x\right)&=\left(C\Gamma_1\left(\mu,\nu\right)+D\Gamma_2\left(\mu,\nu\right)\right)\exp\left(ikx\right)+\left(C\Gamma_3\left(\mu,\nu\right)+D\Gamma_4\left(\mu,\nu\right)\right)\exp\left(-ikx\right)\\&\equiv A\exp\left(ikx\right)+B\exp\left(-ikx\right); \qquad (74)\\ \psi_+\left(x\right)&=C\exp\left(ik'x\right)+D\exp\left(-ik'x\right), \qquad (75)\end{aligned}$$

with C and D integration constants. The functions $\Gamma_i\left(\mu,\nu\right)$ can be found by identifying our Supplementary Equation 74 with equation 11 of [7].

Along these lines, it is important to note that the sign convention employed in [7] is the same as that in Cooper's tutorial [1], and it is analogous to the sign convention employed in the temporal scattering problem for the forward and backward plane waves. In this way, one could expect that relabelling $k\to\omega_0$ and $k'\to N_+\omega_0$ in the expressions of R and T of [7], we would be able to obtain the temporal scattering reflection and transmission coefficients. Unfortunately, this procedure does not allow us to derive closed-form expressions of R and T for the temporal scattering problem because the equations connecting R_1 (T_1) and R_2 (T_2) in S-SUSY and T-SUSY are not analogous. More specifically, note that the asymptotic behaviours of the supersymmetric wave functions in the *spatial scattering problem* are S-SUSY-connected in [1] as:

$$\exp\left(ikx\right)+R_1\exp\left(-ikx\right)=\xi\left(-\frac{\mathrm{d}}{\mathrm{d}x}+W_-\right)\left(\exp\left(ikx\right)+R_2\exp\left(-ikx\right)\right); \tag{76}$$

$$T_1\exp\left(ik'x\right)=\xi\left(-\frac{\mathrm{d}}{\mathrm{d}x}+W_+\right)T_2\exp\left(ik'x\right). \tag{77}$$

In contrast, the asymptotic behaviours of the supersymmetric wave functions in the *temporal scattering problem* are T-SUSY-connected as (we reproduce Supplementary Equations 24 and 25 for clarity):

$$\exp\left(i\omega_0t\right)=\xi\left(-\frac{\mathrm{d}}{\mathrm{d}t}+W_-\right)\exp\left(i\omega_0t\right); \tag{78}$$

$$R_1\exp\left(-iN_+\omega_0t\right)+T_1\exp\left(iN_+\omega_0t\right)=\xi\left(-\frac{\mathrm{d}}{\mathrm{d}t}+W_+\right)\left(R_2\exp\left(-iN_+\omega_0t\right)+T_2\exp\left(iN_+\omega_0t\right)\right). \tag{79}$$

If $R_1=R_2=0$, both systems are analogous and we can perform the previous relabelling to find the temporal scattering reflection and transmission coefficients. For instance, we calculated Supplementary Equation 65 using this procedure. Nevertheless, $R_{1,2}\neq0$ in this case (see Supplementary Figure 11). Consequently, we should recalculate the scattering coefficients for the temporal problem from the beginning.

Considering that $\psi_-\left(t\right)=\exp\left(i\omega_0t\right)$ and $\psi_+\left(t\right)=R\exp\left(-iN_+\omega_0t\right)+T\exp\left(iN_+\omega_0t\right)$, we infer from Supplementary Equations 74 and 75 that $A=1$, $B=0$, $C=T$ and $D=R$. Now, using equation 14 of [7], we find that $C=t_{11}$ and $D=t_{21}$, where the transfer matrix t_{ij} is given by equation 16 of the aforementioned reference. Therefore, performing the transformation of parameters $\alpha=1/2\widetilde{\alpha}$ to match the definition of the hyperbolic step potential of [7] with our definition of the hyperbolic step-index profile (Supplementary Equation 58 taking $\alpha=A=B^{1/2}$), we finally obtain Supplementary Equations 71 and 72.

Supersymmetric time-reversal modulations

Interestingly, an *even* superpotential $W(t) = W(-t)$ allows us to construct T-SUSY time-reversal refractive index profiles of the form $n_{\mathrm{T2}}(t) = n_{\mathrm{T1}}(-t)$ with the same intensity scattering behaviour, certainly an unexpected result taking into account that, in general, $n_{\mathrm{T1}}(-t)$ has different scattering properties from those of the original modulation $n_{\mathrm{T1}}(t)$ (consider, e.g., the step-index case [8]).

Let us analyse this scenario in more detail. To this end, consider a given W with even symmetry. In such a case, we can infer from Riccati's equation that:

$$V_2(t) = W^2(t) + W'(t) = W^2(-t) - W'(-t) = V_1(-t), \tag{80}$$

and then, we find that $n_{\mathrm{T2}}(t) = n_{\mathrm{T1}}(-t)$ by setting $n_{2,-} = n_{1,-}$. Now, rewriting Supplementary Equation 80 as $V_2(\mathbf{a}_1 t) = V_1(\mathbf{a}_2 t)$ and comparing this expression with the SIP condition (Supplementary Equation 32), we find that $M = 0$ and the set of parameters $\mathbf{a}_1$ and $\mathbf{a}_2$ are real numbers related as $a_2 = -a_1 = -1$, i.e., we have a scaling SIP relation between superpartners. In this way, T-SUSY and SIP allow us to find time-reversal refractive index modulations with the same intensity scattering properties.

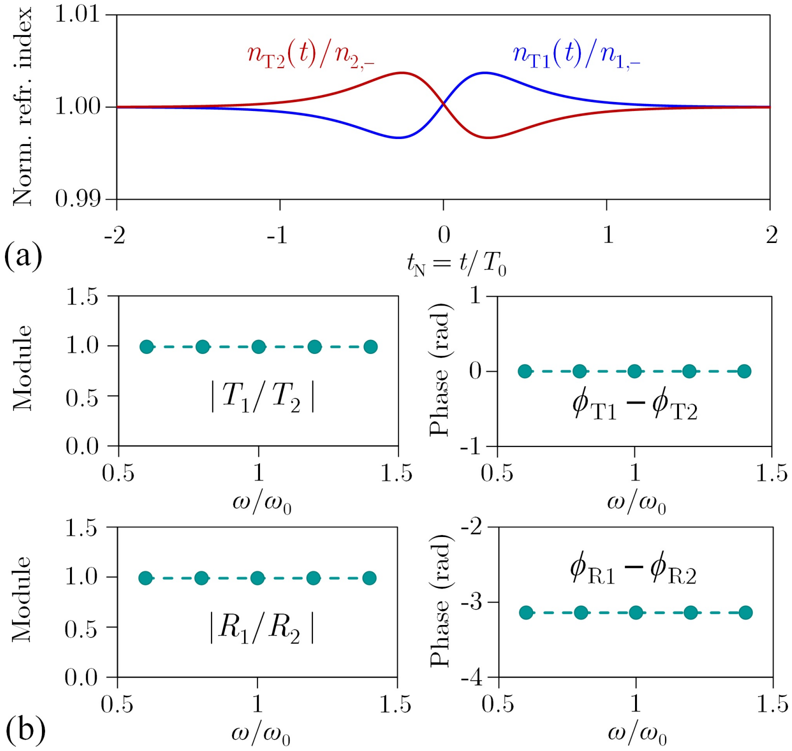

Supplementary Figure 12. (a) Normalised T-SUSY time-reversal refractive index profiles associated with the superpotential of Supplementary Equation 81, and (b) ratios of the scattering coefficients R_1/R_2 and T_1/T_2 associated to the above refractive index profiles (calculated for the case $n_{1,-} = n_{2,-} = 2$) as a function of the ratio ω/ω_0.

As an example, consider an even superpotential of the form:

$$W(t) = B\,\mathrm{sech}(\alpha t), \tag{81}$$

where B and α are real parameters. This superpotential is a particular case of the hyperbolic Scarf II superpotential $W(t) = A\tanh(\alpha t) + B\,\mathrm{sech}(\alpha t)$. The SUSY refractive index profiles $n_{\mathrm{T1,2}}(t)$ can be calculated by combining Riccati's equation and Supplementary Equation 16 bearing in mind that

$\Omega = \omega_0^2 + W_-^2 = \omega_0^2$. The corresponding normalised profiles are shown in Supplementary Figure 12a, and the behaviour of the scattering coefficients as a function of the frequency (numerically calculated from the Supplementary Equation 8) is depicted in Supplementary Figure 12b. Outstandingly, we can see that both time-reversal refractive index profiles have the same intensity scattering properties, not only at $\omega = \omega_0$, but also at $\omega \neq \omega_0$. In other words, the ratios R_1/R_2 and T_1/T_2 have a flat frequency response. In any case, T-SUSY only provides control at the design frequency. We analysed other even superpotentials and we found that R_1/R_2 and T_1/T_2 also presented a flat frequency response. Nevertheless, we cannot extrapolate this singular feature as a general rule in T-SUSY time-reversal modulations. At least, the demonstration is not straightforward.

Supplementary Note 4:
Temporal waveguides – theory

Here, we first detail the theory of unbroken SUSY transformations in temporal waveguides (TWGs) and, secondly, we derive a coupled-mode theory (CMT) for serial TWGs moving with the same speed and direction in a given spatial waveguide.

T-SUSY in temporal waveguides

The unbroken SUSY relation between two quantum-mechanical superpartners $V_{1,2}$ is given by the expression (we set $\hbar^2/2m \equiv 1$ for simplicity) [1]:

$$V_2(x) = V_1(x) - 2\frac{\mathrm{d}^2}{\mathrm{d}x^2}\ln\psi_0^{(1)}(x), \tag{82}$$

where $\psi_0^{(1)}$ is the ground state of V_1. Along this line, note that we can identify an effective potential in equation (7) of the paper of the form:

$$V_i(\tau) \equiv \frac{2}{\beta_2}\beta_{\mathrm{B}i}(\tau). \tag{83}$$

Hence, combining Supplementary Equations 82 and 83 we infer that:

$$\beta_{\mathrm{B}2}(\tau) = \beta_{\mathrm{B}1}(\tau) - \beta_2\frac{\mathrm{d}^2}{\mathrm{d}\tau^2}\ln\psi_0^{(1)}(\tau), \tag{84}$$

where $\psi_0^{(1)}$ is the ground state (fundamental mode) of $\beta_{\mathrm{B}1}$ in this case.

Coupled-mode theory for serial temporal waveguides

Consider two serial TWGs a and b constructed from two different temporal perturbations $\beta_{\mathrm{B},a}(t - z/v_{\mathrm{B}})$ and $\beta_{\mathrm{B},b}(t - z/v_{\mathrm{B}})$ of temporal width $2T_{\mathrm{B},a}$ and $2T_{\mathrm{B},b}$. Furthermore, both TWGs are moving with the same speed v_{B} through the longitudinal z-axis of a given spatial waveguide and are separated T_{ab} in time and $v_{\mathrm{B}}T_{ab}$ in space. Supplementary Figure 13 illustrates this scenario.

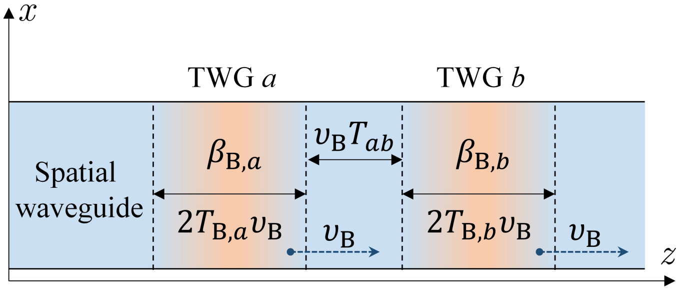

Supplementary Figure 13. Serial temporal waveguides (TWGs) moving with the same speed v_{B} through the longitudinal axis of a given spatial waveguide (blue area).

For the sake of simplicity, let us first assume that both TWGs are operating in the single-mode regime. The fundamental mode of each TWG will be denoted with the subscript a or b. In such a scenario, the complex envelope of the global electric field of this optical structure can be approximated using perturbation theory as ($\tau := t - z/v_{\mathrm{B}}$):

$$A(z,\tau) \simeq \sum_{m=a,b} \mathscr{A}_m(z)\,\psi_m(\tau)\exp\left(i\frac{\Delta\beta_1}{\beta_2}\tau\right)\exp(iK_m z), \tag{85}$$

where $\mathscr{A}_m$ is the complex amplitude of each mode. In isolated conditions (i.e., when each TWG is uncoupled from the other one), we find that $\mathrm{d}\mathscr{A}_m/\mathrm{d}z = 0$ and ψ_m must fulfil the temporal Helmholtz equation (7) of the paper:

$$\left[\frac{\mathrm{d}^2}{\mathrm{d}\tau^2} + 2\frac{K_m}{\beta_2} + \left(\frac{\Delta\beta_1}{\beta_2}\right)^2 - 2\frac{\beta_{\mathrm{B},m}(\tau)}{\beta_2}\right]\psi_m(\tau) = 0. \tag{86}$$

Nonetheless, if the two TWGs are in close proximity (serially coupled), the longitudinal dependence of $\mathscr{A}_m$ accounts for the power exchange between modes (the mode-coupling between temporal modes of serial TWGs takes place through their evanescent tails), ψ_m also fulfils Supplementary Equation 86, and the complex envelope A given by Supplementary Equation 85 must satisfy the time-domain equation [9]:

$$\left(\frac{\partial}{\partial z} + \Delta\beta_1\frac{\partial}{\partial\tau} + i\frac{1}{2}\beta_2\frac{\partial^2}{\partial\tau^2} - i\beta_{\mathrm{B}}(\tau)\right)A(z,\tau) = 0, \tag{87}$$

where $\beta_{\mathrm{B}}(\tau) = \beta_{\mathrm{B},a}(\tau) + \beta_{\mathrm{B},b}(\tau)$. Note that the above equation plays the same role as the wave equation in optical couplers based on parallel spatial waveguides. Hence, substituting Supplementary Equation 85 into Supplementary Equation 87 and using Supplementary Equation 86, we find after some algebra (we omit the independent variables for simplicity):

$$\sum_{m=a,b}\frac{\mathrm{d}\mathscr{A}_m}{\mathrm{d}z}\psi_m\exp(iK_m z) - i(\beta_{\mathrm{B}} - \beta_{\mathrm{B},m})\mathscr{A}_m\psi_m\exp(iK_m z) = 0. \tag{88}$$

From the above equation, we can find the coupled-mode equations describing the power exchange between modes of both serial TWGs. For instance, the coupled-mode equation governing the mode-coupling from mode b to mode a is found: (*i*) multiplying Supplementary Equation 88 by $\psi_a\exp(-iK_a z)$, (*ii*) integrating in $\tau\in(-\infty,\infty)$, and (*iii*) writing the first-order derivative of $\mathscr{A}_{a(b)}$ at the left-hand side. In this way, we obtain:

$$\frac{\mathrm{d}\mathscr{A}_a}{\mathrm{d}z} = ic_a\mathscr{A}_a + \exp(i\Delta K_{b,a}z)\left(i\kappa_{a,b} - \chi_{a,b}\frac{\mathrm{d}}{\mathrm{d}z}\right)\mathscr{A}_b, \tag{89}$$

where $\Delta K_{b,a} := K_b - K_a$. A similar coupled-mode equation describing the coupled power from mode a to mode b can be found by exchanging the subscripts in the above equation. The mode-coupling coefficients (MCCs), accounting for the mode overlapping between ψ_a and ψ_b, are defined as:

$$\chi_{a,b} := \frac{1}{N_a}\int_{-\infty}^{\infty}\psi_b(\tau)\psi_a(\tau)\,\mathrm{d}\tau; \tag{90}$$

$$c_a := \frac{1}{N_a}\int_{-\infty}^{\infty}\beta_{\mathrm{B},b}(\tau)\psi_a^2(\tau)\,\mathrm{d}\tau = \frac{1}{N_a}\int_{\langle 2T_{\mathrm{B},b}\rangle}\beta_{\mathrm{B},b}(\tau)\psi_a^2(\tau)\,\mathrm{d}\tau; \tag{91}$$

$$\kappa_{a,b} := \frac{1}{N_a}\int_{-\infty}^{\infty}\beta_{\mathrm{B},a}(\tau)\psi_b(\tau)\psi_a(\tau)\,\mathrm{d}\tau = \frac{1}{N_a}\int_{\langle 2T_{\mathrm{B},a}\rangle}\beta_{\mathrm{B},a}(\tau)\psi_b(\tau)\psi_a(\tau)\,\mathrm{d}\tau, \tag{92}$$

with $N_a := \int_{-\infty}^{\infty}\psi_a^2(\tau)\,\mathrm{d}\tau$.

It is worthy to note the complete analogy between the CMT of parallel spatial waveguides (see e.g. equation 4.11 in [10] or equation 5 in [11]) and the CMT of serial TWGs (Supplementary Equation 89). In these references, it is demonstrated that $\chi_{a,b}$ can be neglected. However, in serially-coupled TWGs, the MCC $\chi_{a,b}$ is generally higher than the MCCs c_a and $\kappa_{a,b}$ and, therefore, all the MCCs should be retained to guarantee a complete description of the mode-coupling phenomenon. Along this line, note that Supplementary Equation 89 can be rewritten as:

$$\frac{\mathrm{d}\mathscr{A}_a(z)}{\mathrm{d}z} = ic_a^{(\mathrm{eq})}\mathscr{A}_a(z) + i\kappa_{a,b}^{(\mathrm{eq})}\exp(i\Delta K_{b,a}z)\mathscr{A}_b(z), \tag{93}$$

where $c_a^{(\mathrm{eq})} := (c_a - \chi_{a,b}\kappa_{b,a})/(1-\chi_{a,b}\chi_{b,a})$ and $\kappa_{a,b}^{(\mathrm{eq})} := (\kappa_{a,b} - \chi_{a,b}c_b)/(1-\chi_{a,b}\chi_{b,a})$. A similar equation for $\mathrm{d}\mathscr{A}_b(z)/\mathrm{d}z$ can be obtained by exchanging the subscripts a and b in Supplementary Equation 93.

Finally, for completeness, the following considerations are in order:

- If we solve the CMT assuming that only the mode a is excited at $z = 0$ ($\mathscr{A}_b(0) = 0$), we find that:

$$\mathscr{A}_b(z) = i\frac{\kappa_{b,a}^{(\mathrm{eq})}}{\eta}\exp\left(-i\frac{\Delta K_{b,a}}{2}z\right)\sin(\eta z)\,\mathscr{A}_a(0), \tag{94}$$

where:

$$\eta = \sqrt{\kappa_{a,b}^{(\mathrm{eq})}\kappa_{b,a}^{(\mathrm{eq})} + \frac{\left(\Delta K_{b,a} + c_b^{(\mathrm{eq})} - c_a^{(\mathrm{eq})}\right)^2}{4}}. \tag{95}$$

Bearing in mind that $\psi_0^{(2)}$ and $\psi_1^{(1)}$ are degenerate modes in the TPL shown in Fig. 4c of the paper ($\Delta K_{b,a} = 0$), we conclude from Supplementary Equations 94 and 95 that they must exchange their optical power periodically along the z-axis with a coupling length (i.e. the length that maximizes the sinusoidal term of Supplementary Equation 94) $L_\mathrm{C} = \pi/2\eta$ and a coupling efficiency $|\mathscr{A}_b(z = L_\mathrm{C})/\mathscr{A}_a(0)|^2 = \left|\kappa_{b,a}^{(\mathrm{eq})}/\eta\right|^2$.

- In the multi-mode regime, the modes $\{a_n\}_{n=1}^N$ of TWG a exchange optical power with the modes $\{b_n\}_{n=1}^N$ of TWG b. In order to describe this situation, Supplementary Equation 85 must be restated as:

$$A(z,\tau) \simeq \sum_{m=a,b}\sum_{n=1}^{N}\mathscr{A}_{mn}(z)\,\psi_{mn}(\tau)\exp\left(i\frac{\Delta\beta_1}{\beta_2}\tau\right)\exp(iK_{mn}z), \tag{96}$$

and, consequently, the mode-coupling from the modes $\{b_n\}_{n=1}^N$ to mode a_i is governed by the coupled-mode equation:

$$\frac{\mathrm{d}\mathscr{A}_{ai}(z)}{\mathrm{d}z} = ic_{ai}\mathscr{A}_{ai}(z) + \sum_{n=1}^{N}\exp(i\Delta K_{bn,ai}z)\left(i\kappa_{ai,bn} - \chi_{ai,bn}\frac{\mathrm{d}}{\mathrm{d}z}\right)\mathscr{A}_{bn}(z). \tag{97}$$

We cannot observe internal mode-coupling among the modes of a given TWG m if we assume that its temporal perturbation profile $\beta_{\mathrm{B},m}(\tau)$ is invariant during the propagation of the TWG along the longitudinal axis of the spatial waveguide. Nevertheless, in practice, the temporal profile $\beta_{\mathrm{B},m}$ may experience dispersion along the z-axis. The precise longitudinal evolution of $\beta_{\mathrm{B},m}$ depends on the exact physical mechanism used to generate the temporal perturbation in the spatial waveguide. For example, using the XPM with a pump-probe set-up, the shape of the pump pulse will generally be affected by dispersion during propagation [9]. In our case, we could overcome this drawback by selecting the pump wavelength at the zero-dispersion wavelength of the spatial waveguide, provided that the higher-order dispersion terms are negligible. This scenario requires to use, e.g., microstructured optical fibres to tailor the material dispersion properties of the spatial waveguide.

- In serial TWGs with different speed or different propagation directions, the MCCs are found to be space-dependent. Nevertheless, this scenario is out of the scope of this work.

Supplementary Note 5:
Temporal waveguides – numerical analysis

Analogy	Spatial slab (TE modes)	Temporal waveguide
Helmholtz equation	$\left[\frac{\mathrm{d}^2}{\mathrm{d}x^2}+\underbrace{(-\beta_n^2)}_{\Omega_n}-\underbrace{\left(-\frac{\omega^2}{c_0^2}n^2(x)\right)}_{V(x)}\right]\psi_n(x)=0$	$\left[\frac{\mathrm{d}^2}{\mathrm{d}\tau^2}+\underbrace{\frac{2}{\beta_2}K_n+\left(\frac{\Delta\beta_1}{\beta_2}\right)^2}_{\Omega_n}-\underbrace{\frac{2}{\beta_2}\beta_\mathrm{B}(\tau)}_{V(\tau)}\right]\psi_n(\tau)=0$
Waveguide profile	$n(x)=\begin{cases}n_\mathrm{cl} & \lvert x\rvert>a\\ n_\mathrm{co} & \lvert x\rvert\le a\end{cases}$	$\beta_\mathrm{B}(\tau)=\begin{cases}\beta_\mathrm{cl} & \lvert\tau\rvert>T_\mathrm{B}\\ \beta_\mathrm{co} & \lvert\tau\rvert\le T_\mathrm{B}\end{cases}$; $\sqrt{2\beta_2(\beta_\mathrm{cl}-\beta_\mathrm{co})}>\lvert\Delta\beta_1\rvert$
Eigenmodes	$\psi_n(x)=\begin{cases}B\exp\left(-\frac{w_n}{a}(\lvert x\rvert-a)\right) & \lvert x\rvert>a\\ A\cos\left(\frac{u_n}{a}x-\frac{n\pi}{2}\right) & \lvert x\rvert\le a\end{cases}$	$\psi_n(\tau)=\begin{cases}B\exp\left(-\frac{w_n}{T_\mathrm{B}}(\lvert\tau\rvert-T_\mathrm{B})\right) & \lvert\tau\rvert>T_\mathrm{B}\\ A\cos\left(\frac{u_n}{T_\mathrm{B}}\tau-\frac{n\pi}{2}\right) & \lvert\tau\rvert\le T_\mathrm{B}\end{cases}$
Eigenvalue equation	$\nu\sqrt{1-b_n}=\frac{n\pi}{2}+\arctan\left(\sqrt{\frac{b_n}{1-b_n}}\right)$; $n=0,1,2,\ldots$	$\nu\sqrt{1-b_n}=\frac{n\pi}{2}+\arctan\left(\sqrt{\frac{b_n}{1-b_n}}\right)$; $n=0,1,2,\ldots$
Dispersion diagram	$b_n=f(\nu)$	$b_n=f(\nu)$

Supplementary Table 1. Analogy between a step-index dielectric slab waveguide [10] and a step-index temporal waveguide [9]. The constants A and B of the eigenmodes ψ_n are connected by the expression $B=A\cos(u_n-n\pi/2)\,\mathrm{sign}(\tau)^n$, which arises from the continuity boundary condition of the eigenmodes.

Supplementary Table 1 summarises the analogy between a step-index spatial dielectric slab waveguide and a step-index TWG, reported in [9]. Concretely, the analogy only applies to the transversal electric (TE) modes of the slab. As seen, the spatial and temporal Helmholtz equations are analogous with an effective potential V and eigenvalue Ω_n correspondence of the form indicated in Supplementary Table 1. In addition, note that the modal analysis of both structures involves the same eigenvalue equation, where b_n and ν are respectively the normalised phase constant and normalised frequency, given by the expressions:

$$\nu^2=u^2+w^2=\frac{\omega^2}{c_0^2}a^2\left(n_\mathrm{co}^2-n_\mathrm{cl}^2\right)\equiv\frac{2}{\beta_2}T_\mathrm{B}^2\left(\beta_\mathrm{cl}-\beta_\mathrm{co}\right); \tag{98}$$

$$u_n=\nu\sqrt{1-b_n}; \qquad w_n=\nu\sqrt{b_n}; \tag{99}$$

$$b_n=\frac{\beta_n^2/\left(\omega^2/c_0^2\right)-n_\mathrm{cl}^2}{n_\mathrm{co}^2-n_\mathrm{cl}^2}\equiv\frac{\beta_\mathrm{cl}-K_n-(\beta_2/2)\left(\Delta\beta_1/\beta_2\right)^2}{\beta_\mathrm{cl}-\beta_\mathrm{co}}. \tag{100}$$

In the gradual-index case, the analogy is only preserved if the slowly-varying condition of $n(x)$ is satisfied, which requires that $\lvert\delta_x n\rvert\ll\lvert n(x)\rvert$ in $\delta x\sim\lambda_0/\overline{n}$, where $\delta_x n:=n(x+\delta x)-n(x)$, λ_0 is the wavelength in vacuum and $\overline{n}$ is the average value of $n(x)$ in $\delta x=2a$. Otherwise, the TE solutions of the spatial slab, calculated from Maxwell's equations, do not obey the spatial Helmholtz equation depicted in Supplementary Table 1 and, therefore, the analogy is broken.

Hence, in step- and gradual-index TWGs, the eigenfunctions ψ_n and eigenvalues Ω_n of the temporal Helmholtz equation can be calculated from the spatial Helmholtz equation of Supplementary Table 1, provided that the slowly-varying condition of the analogous $n(x)$ profile is satisfied (which is the case in all TWGs analysed in this work). It is important to note that the analogy should be established by selecting values of ω and a that guarantee the same normalised frequency in the spatial slab and in the TWG (Supplementary Equation 98). In this work, we have solved the spatial Helmholtz equation of the analogous slab with CST Microwave Studio. This software allows us to calculate numerically the TE modes of interest directly from Maxwell's equations, which coincide with those obtained from the spatial Helmholtz equation for slowly-varying index profiles $n(x)$, as mentioned above. Once we

calculated the normalised dispersion diagram $b_n = f(\nu)$ of the TE modes with CST, we verified in MATLAB that the corresponding eigenfunctions and eigenvalues fulfil the temporal Helmholtz equation of the TWG under analysis.

Temporal bound states of SUSY TWGs

To complete the information provided by Fig. 4b of the paper, Supplementary Figure 14 shows the spatio-temporal profile of the temporal bound states $\psi_n^{(1,2)}$ supported by both TWGs. As seen in paper Fig. 4b and Supplementary Figure 14, $\psi_0^{(1)}$ has no SUSY counterpart in the eigenvalue spectrum of β_{B2}, i.e., $\psi_0^{(1)}$ is not phase-matched with any temporal bound state $\psi_n^{(2)}$. Contrariwise, the temporal bound states $\psi_{n+1}^{(1)}$ are perfectly phase-matched with the temporal bound states $\psi_n^{(2)}$.

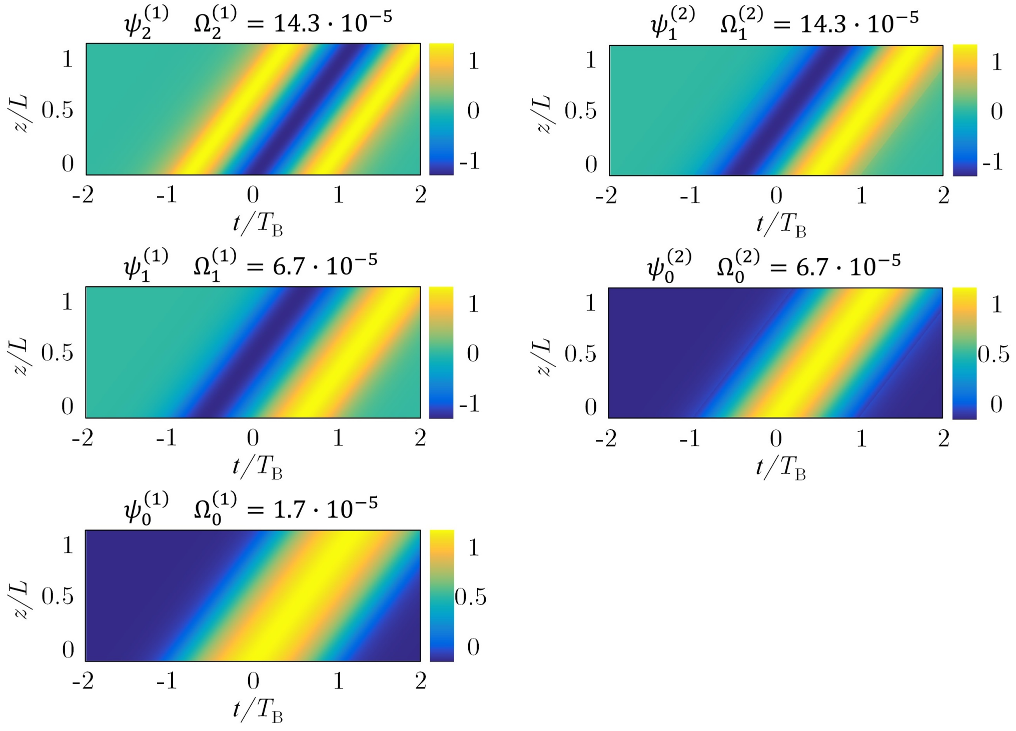

Supplementary Figure 14. Spatio-temporal profile of the eigenfunction $\psi_n^{(1,2)}(t - z/v_{\mathrm{B}})$ and corresponding eigenvalue $\Omega_n^{(1,2)} = 2K_n^{(1,2)}/\beta_2 + \Delta\beta_1^2/\beta_2^2$ in equation (7) of the paper. The temporal and spatial axes are normalised as t/T_{B} and z/L, where L is the length of the spatial waveguide over which the TWGs propagate. Note that v_{B} and L are arbitrary parameters in the numerical simulation. (Colorbar: normalised amplitude).

Note on the numerical analysis of the temporal photonic lantern using the CMT

The numerical simulation shown in Fig. 4d of the main text has been performed in MATLAB by using the CMT. Aimed to facilitate the comprehension of the results, we have taken $L = L_{\mathrm{C}}$ keeping in mind that L (the length of the spatial waveguide over which the TPL propagates) is an arbitrary parameter. Additionally, the parameter L_{C} and the coupling efficiency of the mode conversion can be calculated as indicated on page 25. In particular, the coupling efficiency is 0.96.

Supplementary Note 6: Transmission line – temporal Helmholtz equation

In low-frequency electromagnetism, the so-called time-varying transmission lines (TVTLs), whose effective dielectric properties vary as a function of space and time, constitute an ideal experimental platform to test and exploit all the proposed T-SUSY concepts. TVTLs usually rely on a space- and time-varying capacitance per unit length $C(z,t)$, in which case, the TVTL is governed by a wave equation of the form [12]:

$$\left(\frac{\partial^2}{\partial z^2} - LC(z,t)\frac{\partial^2}{\partial t^2}\right)\Psi(z,t) = 0, \tag{101}$$

where L is the (constant) inductance per unit of length, $\Psi(z,t) := C(z,t)\,v(z,t)$, and v is the TVTL voltage. TVTLs with a space-time variable capacitance can be implemented by periodically loading a transmission line with a bank of distributed varactor diodes [12]. If a modulation of the form $C(z,t) = C_{\mathrm{S}}(z)\,C_{\mathrm{T}}(t)$ is used, expressing the wave function as $\Psi(z,t) = \phi(z)\,\psi(t)$, Supplementary Equation 101 becomes:

$$\frac{1}{C_{\mathrm{S}}(z)}\frac{\phi''(z)}{\phi(z)} = LC_{\mathrm{T}}(t)\frac{\ddot{\psi}(t)}{\psi(t)}. \tag{102}$$

This is satisfied if and only if both sides of the equation are equal to a constant γ. Therefore, we must have:

$$LC_{\mathrm{T}}(t)\frac{\ddot{\psi}(t)}{\psi(t)} = \gamma. \tag{103}$$

Assuming that $C_- := C_{\mathrm{T}}(t \to -\infty)$ is also a constant, we obtain $\gamma = -\omega^2 LC_-$ for a wave with an angular frequency ω at $t \to -\infty$. This leads to the following temporal Helmholtz equation:

$$\left(\frac{\mathrm{d}^2}{\mathrm{d}t^2} + \omega^2\frac{C_-}{C_{\mathrm{T}}(t)}\right)\psi(t) = 0, \tag{104}$$

which exactly matches equation 3 of the main text by defining $N^2(t) := C_-/C_{\mathrm{T}}(t)$. Taking into account that Supplementary Equation 101 is analogous to Supplementary Equation 8 (setting $C_{\mathrm{S}} \equiv 1$), we can identify an *equivalent* time-varying refractive index $n_{\mathrm{T}}(t)$ of the form:

$$\frac{n_{\mathrm{T}}^2(t)}{c_0^2} \equiv LC_{\mathrm{T}}(t)\,. \tag{105}$$

From the above equation, we can infer that the equivalent index background is $n_- = c_0\sqrt{LC_-}$ and the equivalent index excursion is $\Delta n_{\mathrm{T}} = c_0\sqrt{L}\left(\sqrt{C_- + \Delta C_{\mathrm{T}}} - \sqrt{C_-}\right)$. Hence, the normalised index excursion is:

$$\frac{\Delta n_{\mathrm{T}}}{n_-} = \sqrt{1 + \frac{\Delta C_{\mathrm{T}}}{C_-}} - 1. \tag{106}$$

Supplementary Note 7:
Acoustics – temporal Helmholtz equation

In this section, a temporal Helmholtz equation formally equal to equation (3) of the main text is derived for acoustic systems characterised by space- and time-varying properties. Particularly, in the case of pressure acoustics, the spatiotemporal evolution of the acoustic pressure $p\left(\mathbf{r},t\right)$ is governed by the following wave equation [13]:

$$-\frac{\partial}{\partial t}\left(B^{-1}\left(\mathbf{r},t\right)\frac{\partial p\left(\mathbf{r},t\right)}{\partial t}\right)+\nabla\cdot\left(\rho^{-1}\left(\mathbf{r},t\right)\nabla p\left(\mathbf{r},t\right)\right)=0, \tag{107}$$

where the medium bulk modulus $B\left(\mathbf{r},t\right)$ and mass density $\rho\left(\mathbf{r},t\right)$ are, in general, functions of space and time. Considering an acoustic medium for which ρ depends only on time and B depends only on space, it is possible to rewrite Supplementary Equation 107 as:

$$-\frac{\partial^2 p\left(\mathbf{r},t\right)}{\partial t^2}+\frac{B\left(\mathbf{r}\right)}{\rho\left(t\right)}\triangle p\left(\mathbf{r},t\right)=0. \tag{108}$$

Applying separation of variables to the pressure as $p\left(\mathbf{r},t\right)=p_{\mathrm{S}}\left(\mathbf{r}\right)p_{\mathrm{T}}\left(t\right)$, the previous equation can be recast as:

$$\rho\left(t\right)\frac{\ddot{p}_{\mathrm{T}}\left(t\right)}{p_{\mathrm{T}}\left(t\right)}=B\left(\mathbf{r}\right)\frac{\triangle p_{\mathrm{S}}\left(\mathbf{r}\right)}{p_{\mathrm{S}}\left(\mathbf{r}\right)}. \tag{109}$$

Once again, this is satisfied if and only if both sides of the equation are equal to a constant. In analogy with the electromagnetic case, defining $n_{\mathrm{T}}^2\left(t\right):=\rho\left(t\right)$ and assuming that $n_-:=n_{\mathrm{T}}\left(t\rightarrow-\infty\right)$ is also a constant, the following temporal Helmholtz equation is readily obtained:

$$\left(\frac{\mathrm{d}^2}{\mathrm{d}t^2}+\omega^2\frac{n_-^2}{n_{\mathrm{T}}^2\left(t\right)}\right)p_{\mathrm{T}}\left(t\right)=0, \tag{110}$$

that is, equation (3) of the main text. From the previous definitions, the normalised refractive index excursion can be expressed in terms of the normalised density excursion as:

$$\frac{\Delta n_{\mathrm{T}}}{n_-}=\sqrt{1+\frac{\Delta\rho}{\rho}}-1. \tag{111}$$

Acoustic media with a spatial (temporal) dependence of ρ (B) also lead to a temporal Helmholtz equation formally equal to equation (3). In particular, applying separation of variables to $B\left(\mathbf{r},t\right)=B_{\mathrm{S}}\left(\mathbf{r}\right)B_{\mathrm{T}}\left(t\right)$, $\rho\left(\mathbf{r},t\right)=\rho_{\mathrm{S}}\left(\mathbf{r}\right)\rho_{\mathrm{T}}\left(t\right)$, and $p\left(\mathbf{r},t\right)=p_{\mathrm{S}}\left(\mathbf{r}\right)p_{\mathrm{T}}\left(t\right)$ in Supplementary Equation 107, we obtain equation (3) with:

$$\frac{n_-^2}{n_{\mathrm{T}}^2\left(t\right)}:=\rho_-B_-^{-1}\rho_{\mathrm{T}}^{-1}\left(t\right)B_{\mathrm{T}}\left(t\right)+\frac{1}{2\omega^2}\frac{\mathrm{d}^2}{\mathrm{d}t^2}\ln B_{\mathrm{T}}\left(t\right)-\frac{1}{4\omega^2}\left(\frac{\mathrm{d}}{\mathrm{d}t}\ln B_{\mathrm{T}}\left(t\right)\right)^2; \tag{112}$$

$$\psi\left(t\right):=p_{\mathrm{T}}\left(t\right)/\sqrt{B_{\mathrm{T}}\left(t\right)}. \tag{113}$$

where $B_-:=B_{\mathrm{T}}\left(t\rightarrow-\infty\right)$ and $\rho_-:=\rho_{\mathrm{T}}\left(t\rightarrow-\infty\right)$. Note that Supplementary Equation 110 is recovered when $B_{\mathrm{T}}=1$.

Supplementary Note 8:
Elasticity – temporal Helmholtz equation

In this section, a temporal Helmholtz equation formally equal to equation (3) of the main text is derived for the propagation of flexural waves in elastic beams with space- and time-varying properties (other cases could be addressed by applying a similar procedure to the general elastic wave equation). The deflection or transverse motion $w(\mathbf{r},t)$ of the beam is given by the Euler-Bernoulli equation [14,15]:

$$\frac{\partial}{\partial t}\left(m(\mathbf{r},t)\frac{\partial w(\mathbf{r},t)}{\partial t}\right)+\frac{\partial^2}{\partial x^2}\left(D(\mathbf{r},t)\frac{\partial^2 w(\mathbf{r},t)}{\partial x^2}\right)=0, \tag{114}$$

where the stiffness $D(\mathbf{r},t)=EI$ (with E denoting Young's modulus and I the second moment of area of the beam cross section) and the beam linear mass $m(\mathbf{r},t)=\rho A$ (with ρ the mass density and A the cross-sectional area) can be functions of space and time. For a beam with a time-independent mass (which is usually the case) and assuming that the stiffness can be expressed as $D(\mathbf{r},t)=D_{\mathrm{S}}(\mathbf{r})\,D_{\mathrm{T}}(t)$, Supplementary Equation 114 can be rewritten in the following way:

$$\frac{1}{D_{\mathrm{T}}(t)}\frac{\partial^2 w(\mathbf{r},t)}{\partial t^2}=-\frac{1}{m(\mathbf{r})}\frac{\partial^2}{\partial x^2}\left(D_{\mathrm{S}}(\mathbf{r})\frac{\partial^2 w(\mathbf{r},t)}{\partial x^2}\right), \tag{115}$$

Applying separation of variables to the deflection as $w(\mathbf{r},t)=w_{\mathrm{S}}(\mathbf{r})\,w_{\mathrm{T}}(t)$, the previous equation becomes:

$$\frac{1}{D_{\mathrm{T}}(t)}\frac{\ddot{w}_{\mathrm{T}}(t)}{w_{\mathrm{T}}(t)}=-\frac{1}{m(\mathbf{r})\,w_{\mathrm{S}}(\mathbf{r})}\frac{\partial^2}{\partial x^2}\left(D_{\mathrm{S}}(\mathbf{r})\frac{\partial^2 w_{\mathrm{S}}(\mathbf{r})}{\partial x^2}\right). \tag{116}$$

Following the same reasoning as in Supplementary Notes 1, 6 and 7, and defining $n_{\mathrm{T}}^2(t):=1/D_{\mathrm{T}}(t)$ with the index background $n_-:=n_{\mathrm{T}}(t\to-\infty)$ being a constant, we obtain again a temporal Helmholtz equation of the form:

$$\left(\frac{\mathrm{d}^2}{\mathrm{d}t^2}+\omega^2\frac{n_-^2}{n_{\mathrm{T}}^2(t)}\right)w_{\mathrm{T}}(t)=0. \tag{117}$$

In this case, the normalised refractive index excursion expressed in terms of the normalised density excursion $\alpha:=\Delta D_{\mathrm{T}}/D_-$ reads:

$$\frac{\Delta n_{\mathrm{T}}}{n_-}=\left|\frac{1}{\sqrt{1+\alpha}}-1\right|. \tag{118}$$

Finally, we would like to point out that, while T-SUSY is a natural underlying property of the Euler-Bernoulli equation, S-SUSY is not, as the spatial derivative that appears in this equation is of fourth order.

Supplementary References

[1] Cooper, F., Khare, A. & Sukhatme, U. Supersymmetry and quantum mechanics. *Phys. Rep.* **251,** 267 (1995).

[2] Midya, B. Supersymmetry-generated one-way-invisible PT-symmetric optical crystals. *Phys. Rev. A* **89,** 032116 (2014).

[3] Farkas, B. & Wegner, S.-A. Variations on Barbalat's lemma. Preprint at arxiv.org/abs/1411.1611 (2014).

[4] Vázquez, J. M., Mazilu, M., Miller, A. & Galbraith, I. Wavelet transforms for optical pulse analysis. *J. Opt. Soc. Am. A* **22,** 2890-2899 (2005).

[5] Dantus, M. & Lozovoy, V. V. Experimental coherent laser control of physicochemical processes. *Chem. Rev.* **104,** 1813-1860 (2004).

[6] Weiner, A. Ultrafast optical pulse shaping: a tutorial review. *Optics Communications* **284,** 3669-3692 (2011).

[7] Gadella, M., Kurub, Ş. & Negroa, J. The hyperbolic step potential: anti-bound states, SUSY partners and Wigner time delays. *Annals of Physics* **379,** 86-101 (2017).

[8] Mendonça, J. T. & Shukla, P. K. Time refraction and time reflection: two basic concepts. *Physica Scripta* **65,** 160-163 (2002).

[9] Plansinis, B. W., Donaldson, W. R. & Agrawal, G. P. Temporal waveguides for optical pulses. *J. Opt. Soc. Am. B* **33,** 1112-1119 (2016).

[10] Okamoto, K. *Fundamentals of Optical Waveguides.* 2nd ed. (Elsevier, Burlington, 2006).

[11] Macho, A., Morant, M. & Llorente, R. Unified model of linear and nonlinear crosstalk in multi-core fiber. *J. Lightwave Technol.* **34,** 3035-3046 (2016).

[12] Qin, S., Xu, Q. & Wang, Y. E. Nonreciprocal components with distributedly modulated capacitors. *IEEE Transactions on Microwave Theory and Techniques* **62**, 2260-2272 (2014).

[13] Landau, L. D. & Lifshitz, L. M. *Fluids Mechanics: Course of Theoretical Physics.* (Pergamon Press, Oxford, 1959).

[14] Gere, J. M. & Timoshenko, S. P. *Mechanics of Materials.* (PWS Publishing Company, Boston, 1997).

[15] Trainiti, G., Xia, Y., Marconi, J., Cazzulani, G., Erturk, A. & Ruzzene, M. Time-periodic stiffness modulation in elastic metamaterials for selective wave filtering: theory and experiment. *Phys. Rev. Lett.* **122**, 124301 (2019).
