# Supplementary material for: Supersymmetry in the time domain and its applications in optics
Source: Nat Commun. 2020 Feb 10;11:813. doi: 10.1038/s41467-020-14634-0 (PMC7010821; doi:10.1038/s41467-020-14634-0)
Supplement: Supplementary file 3 — Description of Additional Supplementary Files [file 41467_2020_14634_MOESM3_ESM.pdf]

Description of Additional Supplementary Files

File name: Supplementary movie 1
Description: Supplementary movie 1 shows an example of spatial reflection for an optical pulse going through a time-invariant spatial (i.e. heterogeneous) step-index medium.

File name: Supplementary movie 2
Description: Supplementary movie 2 shows an example of temporal reflection for an optical pulse going through a time-varying non-spatial (i.e. homogeneous) step-index medium.

File name: Supplementary movie 3
Description: Supplementary movie 3 shows the pulse propagation evolution at $\omega = \omega 0$ through the reflectionless time-varying medium with normalised index profile nT2 $(t)/n2,-$ shown in Fig. 2b of the paper. The movie corresponds with Fig. 2d of the paper.

File name: Supplementary movie 4
Description: Supplementary movie 4 shows the pulse propagation evolution at $\omega = \omega 0$ through the reflectionless time-varying medium with normalised index profile nT6 $(t)/n6,-$ depicted in Fig. 3b of the paper.

File name: Supplementary movie 5
Description: Supplementary movie 5 shows the pulse shape transformation performed in a temporal photonic lantern. The pulse shape transformation results from the energy transfer between the temporal bound states $\psi 0$ (2) and $\psi 1$ (1) of the temporal waveguides over which the temporal photonic lantern is constructed.
