# Supplementary figures and images for: Supersymmetry in the time domain and its applications in optics
Source: Nat Commun. 2020 Feb 10;11:813. doi: 10.1038/s41467-020-14634-0 (PMC7010821; doi:10.1038/s41467-020-14634-0)

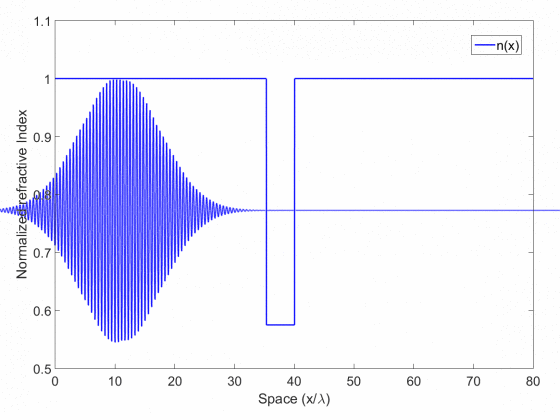

Supplement: Supplementary file 4 — 1.Step spatial scattering [file 41467_2020_14634_MOESM4_ESM.gif]

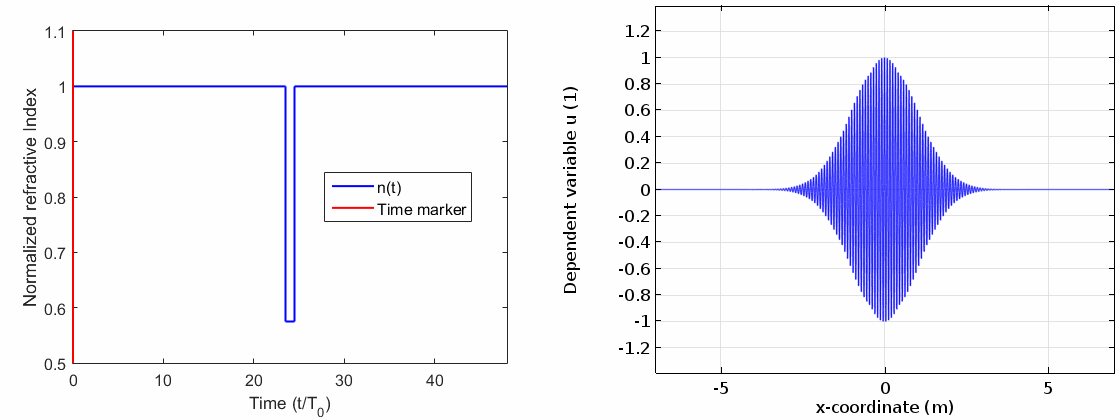

Supplement: Supplementary file 5 — 2.Step temporal scattering [file 41467_2020_14634_MOESM5_ESM.gif]

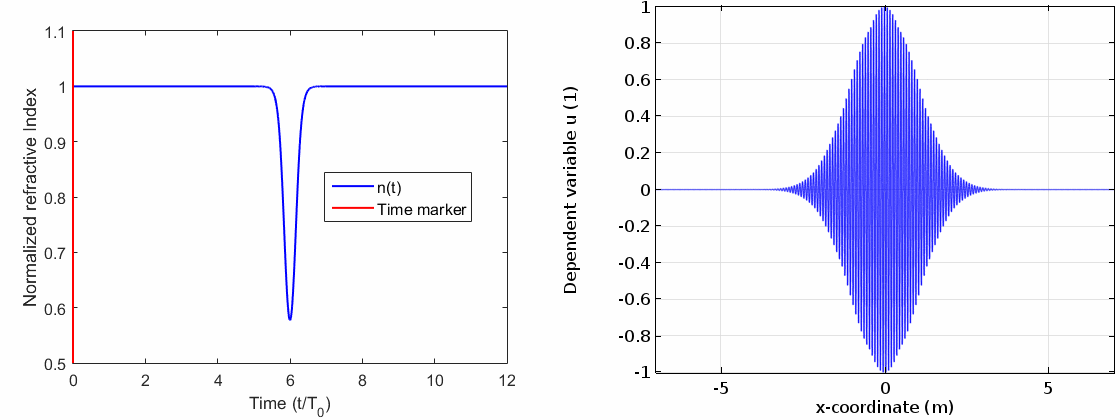

Supplement: Supplementary file 6 — 3.Hyperbolic secant modulation [file 41467_2020_14634_MOESM6_ESM.gif]

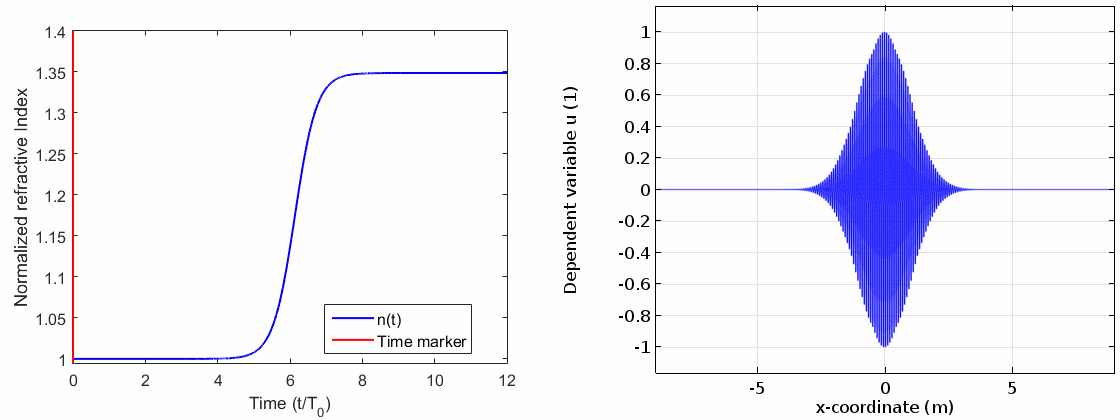

Supplement: Supplementary file 7 — 4.Hyperbolic step modulation [file 41467_2020_14634_MOESM7_ESM.gif]

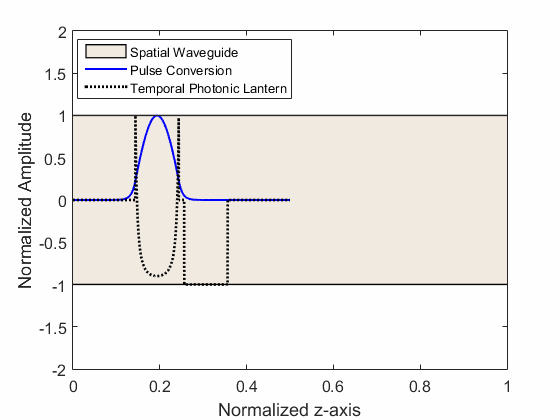

Supplement: Supplementary file 8 — 5.Temporal photonic lantern [file 41467_2020_14634_MOESM8_ESM.gif]
